# Donor Atom Preference of Organoruthenium and Organorhodium Cations on the Interaction with Novel Ambidentate (N,N) and (O,O) Chelating Ligands in Aqueous Solution

**DOI:** 10.3390/molecules26123586

**Published:** 2021-06-11

**Authors:** Sándor Nagy, András Ozsváth, Attila Cs. Bényei, Etelka Farkas, Péter Buglyó

**Affiliations:** 1 Department of Inorganic and Analytical Chemistry, University of Debrecen, H-4032 Debrecen, Hungary; nagy.sandor@science.unideb.hu (S.N.); ozsvath.andras92@gmail.com (A.O.); efarkas@science.unideb.hu (E.F.); 2 Department of Physical Chemistry, University of Debrecen, H-4032 Debrecen, Hungary; benyei.attila@science.unideb.hu

**Keywords:** organoruthenium, organorhodium, palladium, anticancer, multichelating ligand, complex, speciation

## Abstract

Two novel, pyridinone-based chelating ligands containing separated (O,O) and (N_amino_,N_het_) chelating sets (N_amino_ = secondary amine; N_het_ = pyrrole N for H(L3) (1-(3-(((1H-pyrrole-2-yl)methyl)-amino)propyl)-3-hydroxy-2-methylpyridin-4(1H)-one) or pyridine N for H(L5) (3-hydroxy-2-methyl-1-(3-((pyridin-2-ylmethyl)amino)propyl)pyridin-4(1H)-one)) were synthesized via reduction of the appropriate imines. Their proton dissociation processes were explored, and the molecular structures of two synthons were assessed by X-ray crystallography. These ambidentate chelating ligands are intended to develop Co(III)/PGM (PGM = platinum group metal) heterobimetallic multitargeted complexes with anticancer potential. To explore their metal ion binding ability, the interaction with Pd(II), [(η^6^-*p*-cym)Ru]^2+^ and [(η^5^-Cp*)Rh]^2+^ (*p*-cym = 1-methyl-4-isopropylbenzene, Cp* = pentamethyl-cyclopentadienyl anion) cations was studied in aqueous solution with the combined use of pH-potentiometry, NMR and HR ESI-MS. In general, organorhodium was found to form more labile complexes over ruthenium, while complexation of the (N,N) chelating set was slower than the processes of the pyridinone unit with (O,O) coordination. Formation of the organoruthenium complexes starts at lower pH (higher thermodynamic stabilities of the corresponding complexes) than for [(η^5^-Cp*)Rh]^2+^ but, due to the higher affinity of [η^6^-*p*-cym)Ru]^2+^ towards hydrolysis, the complexed ligands are capable of competing with hydroxide ion in a lesser extent than for the rhodium systems. As a result, under biologically relevant conditions, the rhodium binding effectivity of the ligands becomes comparable or even slightly higher than their effectivity towards ruthenium. Our results indicate that H(L3) is a less efficient (N,N) chelator for these metal ions than H(L5). Similarly, due to the relative effectivity of the (O,O) and (N,N) chelates at a 1:1 metal-ion-to-ligand ratio, H(L5) coordinates in a (N,N) manner to both cations in the whole pH range studied while, for H(L3), the complexation starts with (O,O) coordination. At a 2:1 metal-ion-to-ligand ratio, H(L3) cannot hinder the intensive hydrolysis of the second metal ion, although a small amount of 2:1 complex with [(η^5^-Cp*)Rh]^2+^ can also be detected.

## 1. Introduction

In addition to a large number of organic molecules, square planar Pt(II) complexes are also widely used in the chemotherapy of various cancers [1,2,3]. Although the properties of metal complexes can be tailored significantly by changing numerous parameters (e.g., size, charge, geometry, lipophilic/hydrophilic character, thermodynamic stability, kinetic behavior of the complexes; hard–soft character of the central metal ion and the coordinating donor atoms), all the clinically applied Pt(II)-based drugs suffer from lack of selectivity resulting in serious side effects and development of resistance during the treatment [2,3,4,5]. To circumvent this problem, one direction can be the development of highly selective, low-concentration, effective metal complexes with much fewer side effects. This might be achieved by considering multitargeted compounds capable of acting on more than one biological target.

Hypoxia present in cancer tissues is a remarkable difference from normoxic conditions in healthy, untransformed cells enabling the development of metal complexes that are selectively activated under hypoxic conditions. Due to the large difference, in general, in the stability and in the rate of ligand exchange reactions, cobalt complexes can be promising candidates for selective hypoxia-activation [6,7,8,9,10]. The inert Co(III) form can act namely as a chaperon carrying a biologically active ligand, and in the more reductive environment of the cancer tissue via selective reduction and subsequent dissociation of the Co(II) species formed, targeted release of the active biomolecule can be achieved. With the use of ambidentate chelating ligands, in addition to Co(III), a second metal ion with proven anticancer potential (e.g., platinum group metal (PGM) ions, such as Pt(II), half-sandwich type ruthenium(II) od rhodium(III)) can also be incorporated into the molecule resulting in heterobimetallic complexes [11,12]. To achieve metal ion selectivity, ambidentate ligands having chelating donor atom sets with different hard–soft characteristics are desired. During the design of these bimetallic complexes, the preference of the hard Co(III) toward an (O,O) chelate while that of the soft PGM ions toward an (N,N) chelate is expected. However, detailed solution equilibrium studies may provide valuable information on the issue. With the aid of the explored trends on the donor atom preference, stability of the chelates formed and the kinetic behavior, rational design of novel chelators with separate metal ion binding sites and synthesis of their bimetallic Co/PGM complexes can also be enhanced.

Recently we have reported on the Pd(II) (as a Pt(II) model but with faster ligand exchange properties) binding capabilities of various primary and secondary di- and tripeptide hydroxamates [11]. The results indicated that 4N and 3N coordinated mononuclear complexes were formed with the tri- and dipeptide derivatives, respectively. Moreover, these ligands were able to bind a metal ion excess via their free hydroxamate groups. For the secondary tripeptide hydroxamic acid, the coordination sphere of the metal ion was saturated by (NH_2_, N_amide_, N_amide_, O_hydr._) donors, hindering the formation of further dinuclear species. Notably, in addition to the complexation processes, Pd(II)-assisted hydrolysis of the hydroxamic group of the ligands was detected in a ligand, as well as a binding-mode-dependent manner. No hydrolysis was found in the 4N-donor primary tripeptide hydroxamic acid containing system, slow hydrolysis of the ligands was detected with the 3N donor L-AlaGlyGlyN(Me)OH and L-Ala-L-AlaNHOH, while very fast hydrolysis of the 2N-donor secondary dipeptide hydroxamic acid occurred. In all systems, Pd(II) was reduced to Pd(0) by the corresponding hydroxylamine formed in the hydrolytic reactions [11].

Due to the non-innocent character of the hydroxamate function toward Pd(II), we have replaced it by a pyridinone entity with a (O,O) chelating set too [12]. Our results indicated that the novel peptide conjugate is capable of binding even two equivalents of Pd(II) in solution forming [Pd_2_H_–n_L] (*n* = 1–4) type species with the involvement both of the (O,O) and (N,N) donor sets over a wide pH range. At a 1:1 ratio, however, the expected coordination of the metal ion to the peptide backbone of the ligand is demonstrated, resulting in the formation of N-coordinated species with the involvement of an increasing number of peptide nitrogens beside the anchoring terminal amino group increasing pH. NMR indicated the peptide conjugate to react with [Co(tren)]^3+^ (tren = tris(2-aminoethyl)amine), resulting in the exclusive formation of the (O,O) coordinated Co(III) complex and, upon addition of Pd(II), the formation of the desired Co/Pd complex. Isolation of a 2N coordinated Pd(II) complex in which the remaining coordination sites of the metal ion are taken by two chloride ions was also done, giving rise to the synthesis of bimetallic complexes incorporating the desired Co(III) entity by using the free (O,O) part of the molecule [12].

Complexes bearing the [(η^6^-*p*-cym)Ru]^2+^ or [(η^5^-Cp*)Rh]^2+^ (*p*-cym = 1-methyl-4-isopropylbenzene; Cp* = pentamethyl-cyclopentadienyl anion) entities play an important role as promising candidates with—depending upon the coordinating ligands—proven anticancer potential. Regarding the organoruthenium and –rhodium solution equilibrium studies, complexation of these cations with ambidentate type aminohydroxamates [13,14], peptidehydroxamates [15], histidine-containing oligopeptides [16,17], an 1,10-phenanthroline conjugated SAHA (SAHA = suberoylanilide hydroxamic acid) [18], an 8-hydroxy-quinoline-proline hybrid [19] and with 2,4-dipicolinic acid [20] was already explored, too.

The aim of the present work was the synthesis and characterization of novel ambidentate ligands containing only two N donors to coordinate besides the above pyridinone-based (O,O) chelating set and to explore the Pd(II), [(η^6^-*p*-cym)Ru]^2+^ and [(η^5^-Cp*)Rh]^2+^ binding capabilities in aqueous solution. This enables understanding the effect of important factors, e.g., type and basicity of the N-donors. Shedding light on these interactions may provide useful information regarding the successful synthesis of Co(III)/PGM heterobimetallic complexes, exploring the donor atom preference of these organometallic metal ions, and, furthermore, about the fate of these administered complexes with anticancer potential after hypoxia activation.

**Figure 1:**
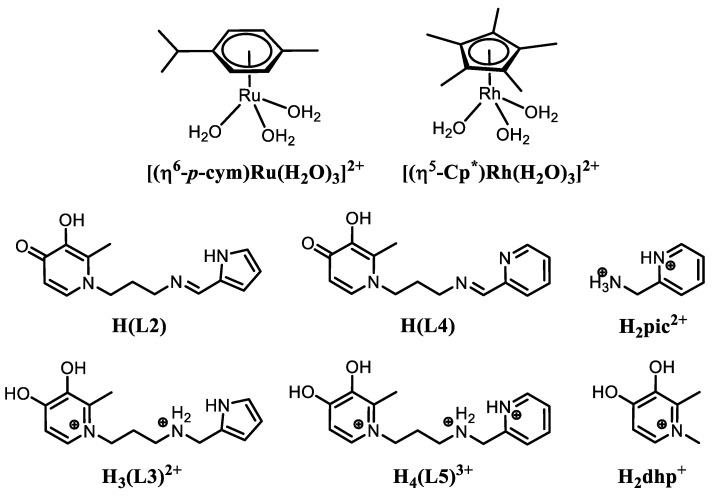
Structure of the studied metal ions, ([(η^6^-*p*-cym)Ru(H_2_O)_3_]^2+^ and [(η^5^-Cp*)Rh(H_2_O)_3_]^2+^), imine ligands, H(L2) and H(L4), as well as the fully protonated forms of their reduced counterparts H_3_(L3)^2+^ and H_4_(L5)^3+^ together with some model ligands, H_2_pic^2+^ and H_2_dhp^+^.

Herein, we report on the synthesis of two novel imines (HL(2) and HL(4), Figure 1 together with the synthesis, characterization and acid-base properties of their reduced counterparts (HL(3) and HL(5), as shown in Figure 1. A detailed solution equilibrium study on the [(η^6^-*p*-cym)Ru]^2+^– and [(η^5^-Cp*)Rh]^2+^–L3 and –L5 systems was also carried out by the combined use of pH-potentiometry, NMR and HR-ESI-TOF-MS to determine the stability of the various species formed, together with their most likely binding modes and to explore the pH-dependent donor atom preference of the half-sandwich type organometallic cations toward the ambidentate H(L3) and H(L5) ligands that may assist in developing Co/PGM heterobimetallic complexes. The study also enabled to explore the effect of the type and basicity of the N-donors of the ligands and the effect of the different hydrolytic and kinetic behaviour of the metal ions on the complexation processes.

## 2. Experimental

### 2.1. Materials and Methods

**Scheme 1:**
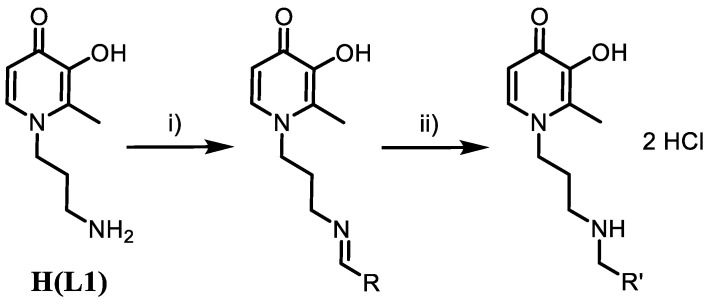
Synthesis of the ligands (H(L2) (R = pyrrole); H(L3)·2 HCl (R’ = pyrrole); H(L4) (R = pyridine); H(L5)·2 HCl (R’ = pyridine)). Reagents and conditions: (**i**) abs. EtOH, pyrrole-2-carboxaldehyde or pyridine-2-carboxaldehyde, 85 °C, ~2 h; (**ii**) MeOH, NaBH_4_, 0 °C (~2 h), RT overnight, 6M HCl, abs. EtOH.

1,3-diaminopropane, maltol, pyrrole-2-carboxaldehyde, 2-pyridinecarboxaldehyde, NaBH_4_, [(η^5^-Cp*)RhCl_2_]_2_, [(η^6^-*p*-cym)RuCl_2_]_2_, KOH, KNO_3_, KCl, MeOH, EtOH, Et_2_O, D_2_O and *d*_6_-DMSO were all commercial products from Acros Organics, Aldrich, Molar and Strem Chemicals. 1-(3-aminopropyl)-3-hydroxy-2-methylpyridin-4(1H)-one, H(L1) (Scheme 1), was prepared according to a method in the literature [21]. Aqueous solutions of the half-sandwich cations were obtained from the respective chloride-bridged precursors by the removal of chloride ions using an equivalent amount of silver nitrate. Pd(II) stock solutions were prepared from K_2_[PdCl_4_] (Sigma-Aldrich, St. Louis, MO, USA) by using doubly deionized and ultrafiltered water from a Milli-Q RG (Millipore, Burlington, MA, USA) water purification system.

### 2.2. Synthesis of the Ligands

*1-(3-(((1H-pyrrole-2-yl)methylene)amino)propyl)-3-hydroxy-2-methylpyridin-4(1H)-one* (H(L2)). Pyrrole-2-carboxaldehyde (1.04 g, 10.94 mmol) was added to H(L1) (2.03 g, 10.91 mmol) dissolved in 100 mL abs. EtOH. The solution was stirred at 85 °C for 2 h and concentrated to 5 mL. On cooling at 4 °C, the title compound appeared as a pale yellow powder. Yield: 2.44 g (86%). ^1^H NMR (400 MHz, *d*_6_-DMSO): δ/ppm = 11.37 (s, 1H, -NH); 8.09 (s, 1H, -CH); 7.61 (d, 1H, pyridinone H); 6.90 (s, 1H, pyrrole H); 6.45 (d, 1H, pyrrole H); 6.11 (m, 2H, pyridinone and pyrrole H); 4.04 (t, 2H, -CH_2_); 3.44 (t, 2H, -CH_2_); 2.29 (s, 3H, -CH_3_); 1.90 (p, 2H, -CH_2_). ^13^C NMR (100 MHz, *d*_6_-DMSO): δ/ppm = 168.86 (pyridinone C = O), 152.32 (-N=CH), 145.56 (pyridinone C-OH), 137.76 (pyridinone -CH), 129.96 (pyrrole -C), 128.57 (pyridinone -C), 122.14 (pyrrole -CH), 113.50 (pyrrole -CH), 110.57 (pyridinone -CH), 108.90 (pyrrole -CH), 56.41 (-CH_2_), 50.81 (-CH_2_), 31.94 (-CH_2_), 11.36 (-CH_3_). IR (KBr)/cm^–1^: 3160 (*υ*_O-H_), 2977 (*υ*_C-H_), 2842 (*υ*_C-H_), 1637(*υ*_C=O_), 1561 (*υ*_C=N_), 1509 (*υ*_C=C_), 1234 (*υ*_C-O_). Anal. Required for C_14_H_17_N_3_O_2_·0.2EtOH: C, 64.41, H, 6.83, N, 15.65%. Found: C, 64.52, H, 7.11, N, 16.01%. MS (ESI, positive ion): *m/z* 557.2273 [2(H(L2)) + K]^+^ (calc. 557.2273); 541.2534 [2(H(L2)) + Na]^+^ (calc. 541.2534); 519.2717 [2(H(L2)) + H]^+^ (calc. 519.2714); 298.0953 [H(L2) + K]^+^ (calc. 298.0952); 282.1213 [H(L2) + Na]^+^ (calc. 282.1213); 260.1398 [H(L2) + H]^+^ (calc. 260.1394).

*1-(3-(((1H-pyrrole-2-yl)methyl)amino)propyl)-3-hydroxy-2-methylpyridin-4(1H)-one·2HCl* (H (L3)·2 HCl). H(L2) (1.01 g, 3.90 mmol) was dissolved in 100 mL MeOH. After cooling in ice NaBH_4_ (0.39 g, 10.31 mmol) was added to the solution in small portions. The mixture was stirred overnight at room temperature. The pH of the solution was set to 2 with 6M HCl and evaporated to dryness. The crude product was dissolved in 10 mL abs. EtOH and filtered. After filtration, the solvent was removed, and the resulting oil was extracted with 3 × 10 mL of diethylether. Removal of the solvent made the title compound a pale yellow powder. Yield: 0.77 g (59%). ^1^H NMR (400 MHz, D_2_O): δ/ppm = 8.04 (d, 1H, pyridinone H); 7.14 (d, 1H, pyridinone H); 6.94 (dd, 1H, pyrrole H); 6.34 (dd, 1H, pyrrole H); 6.23 (t, 1H, pyrrole H); 4.42 (t, 2H, -CH_2_); 4.28 (s, 2H, -CH_2_); 3.10 (t, 2H, -CH_2_); 2.58 (s, 3H, -CH_3_); 2.20 (p, 2H, -CH_2_). ^1^H NMR (400 MHz, *d*_6_-DMSO): δ/ppm = 11.13 (s, 1H, -NH); 9.58 (s, 2H, -NH_2_); 8.31 (d, 1H, pyridinone H); 7.34 (d, 1H, pyridinone H); 6.83 (dd, 1H, pyrrole H); 6.19 (t, 1H, pyrrole H); 6.01 (dd, 1H, pyrrole H); 4.49 (t, 2H, -CH_2_); 4.09 (t, 2H, -CH_2_); 2.88 (s, 2H, -CH_2_); 2.54 (s, 3H, -CH_3_); 2.15 (p, 2H, -CH_2_). ^13^C NMR (100 MHz, *d*_6_-DMSO): δ/ppm = 158.68 (pyridinone C=O), 143.10 (pyridinone C-OH), 141.60 (pyridinone -C), 138.05 (pyridinone -CH), 121.49 (pyrrole -C), 119.24 (pyrrole -CH), 110.93 (pyridinone -CH), 110.24 (pyrrole -CH), 107.91 (pyrrole -CH), 53.16 (-CH_2_), 42.70 (-CH_2_), 42.08 (-CH_2_), 25.98 (-CH_2_), 12.69 (-CH_3_). IR (KBr)/cm^–1^: 3256 (*υ*_O–H_), 2785 (*υ*_N–H_), 1633 (*υ*_C=O_), 1508 (*υ*_C=C_), 1338 (*υ*_C–N_), 1253 (*υ*_C–O_). Anal. Required for C_14_H_19_N_3_O_2_·2HCl·1.5H_2_O: C, 46.55, H, 6.70, N, 11.63%. Found: C, 46.76, H, 6.97, N, 11.47%. MS (ESI, positive ion): *m/z* 262.1551 [H(L3) + H]^+^ (calc. 262.1550); 183.1129 [C_9_H_15_N_2_O_2_]^+^ (calc. 183.1134).

*3-hydroxy-2-methyl-1-(3-((pyridin-2-ylmethylene)amino)propyl)pyridin-4(1H)-one (H(L4)).* Pyridine-2-carboxaldehyde (540 µL, 5.68 mmol) was added to H(L1) (1.05 g, 5.76 mmol) dissolved in 100 mL abs. EtOH. The solution was stirred at 85 °C for 2 h. The solvent was removed, and the crude yellow oil was dried in vacuo and extracted with 4 × 10 mL of diethylether. Removal of the solvent made the title compound a pale yellow powder. Yield: 0.94 g (60%). ^1^H NMR (400 MHz, *d*_6_-DMSO): δ/ppm = 8.64 (d, 1H, pyridine H); 8.38 (s, 1H, CH); 7.97 (d, 1H, pyridine H); 7.88 (t, 1H, pyridine H); 7.60 (d, 1H, pyridinone H); 7.46 (t, 1H, pyridine H); 6.12 (d, 1H, pyridinone H); 4.06 (t, 2H, -CH_2_); 3.65 (t, 2H, -CH_2_); 2.31 (s, 3H, -CH_3_); 2.01 (p, 2H, -CH_2_). ^13^C NMR (100 MHz, *d*_6_-DMSO): δ/ppm = 168.85 (pyridinone -C=O), 162.42 (-N=CH), 154.03 (pyridine -C), 149.35 (pyridine -CH), 145.53 (pyridinone -C-OH), 137.63 (pyridinone -CH), 136.88 (pyridine -CH), 128.51 (pyridinone -C), 125.17 (pyridine -CH), 120.47 (pyridine -CH), 110.60 (pyridinone -CH), 56.77 (-CH_2_), 50.87 (-CH_2_), 31.46 (-CH_2_), 11.33 (-CH_3_). IR (KBr)/cm^–1^: 3154 (*υ*_O–H_), 1624 (*υ*_C=O_), 1579 (*υ*_C=N_), 1508 (*υ*_C=C_), 1245 (*υ*_C–O_). Anal. Required for C_15_H_17_N_3_O_2_·0.15EtOH: C, 66.05, H, 6.48, N, 15.10%. Found: C, 66.17, H, 6.46, N, 14.97%. MS (ESI, positive ion): *m/z* 581.2261 [2(H(L4)) + K]^+^ (calc. 581.2273); 565.2531 [2(H(L4)) + Na]^+^ (calc. 565.2532); 310.0952 [H(L4) + K]^+^ (calc. 310.0952); 294.1214 [H(L4) + Na]^+^ (calc. 294.1213); 272.1393 [H(L4) + H]^+^ (calc. 272.1394).

*3-hydroxy-2-methyl-1-(3-((pyridin-2-ylmethyl)amino)propyl)pyridin-4(1H)-one·2HCl* (H(L5)·2 HCl). It was synthesized analogously to H(L3)·2 HCl using H(L4) (0.94 g, 3.45 mmol), 90 mL MeOH and NaBH_4_ (0.37 g, 9.81 mmol). Pale yellow powder. Yield: 0.92 g (77%). ^1^H NMR (400 MHz, D_2_O): δ/ppm = 8.71 (d, 1H, pyridine H); 8.23 (dt, 1H, pyridine H); 8.09 (d, 1H, pyridinone H); 7.81 (d, 1H, pyridine H); 7.74 (dt, 1H, pyridine H); 7.15 (d, 1H, pyridinone H); 4.54 (s, 2H, -CH_2_); 4.48 (t, 2H, -CH_2_); 3.32 (t, 2H, -CH_2_); 2.62 (s, 3H, -CH_3_); 2.35 (p, 2H, -CH_2_).^1^H NMR (400 MHz, *d_6_*-DMSO): δ/ppm = 10.01 (s, 2H, -NH_2_); 8.68 (d, 1H, pyridine H); 8.37 (d, 1H, pyridinone H); 8.02 (t, 1H, pyridine H); 7.76 (d, 1H, pyridine H); 7.54 (t, 1H, pyridine H); 7.44 (d, 1H, pyridinone H); 4.54 (t, 2H, -CH_2_); 4.35 (s, 2H, -CH_2_); 3.04 (s, 2H, -CH_2_); 2.57 (s, 3H, -CH_3_); 2.26 (p, 2H, -CH_2_). ^13^C NMR (100 MHz, *d*_6_-DMSO): δ/ppm = 158.62 (pyridinone -C=O), 149.80 (pyridine -C), 146.11 (pyridine -CH), 143.08 (pyridinone -C-OH), 141.88 (pyridinone -C), 141.16 (pyridine -CH), 138.11 (pyridinone -CH), 125.86 (pyridine -CH), 125.65 (pyridine -CH), 110.87 (pyridinone -CH), 53.29 (-CH_2_), 48.55 (-CH_2_), 43.60 (-CH_2_), 26.01 (-CH_2_), 12.76 (-CH_3_). IR (KBr)/cm^–1^: 2993 (*υ*_O–H_), 2717 (*υ*_N–H_), 1633 (*υ*_C=O_), 1504 (*υ*_C=C_), 1335 (*υ*_C–N_), 1229 (*υ*_C–O_). Anal. Required for C_15_H_19_N_3_O_2_·2HCl·0.4H_2_O: C, 50.97, H, 6.22, N, 11.89%. Found: C, 51.25, H, 6.47, N, 11.87%. MS (ESI, positive ion): *m/z* 274.1554 [H(L5) + H]^+^ (calc. 274.1550); 183.1134 [C_9_H_15_N_2_O_2_]^+^ (calc. 183.1134).

### 2.3. Solution Studies

For solution studies, doubly deionized and ultra-filtered water produced by a Milli-Q RG (Millipore) water purification system was used. The pH-potentiometric measurements were carried out at an ionic strength of 0.20 M KCl and at 25.0 ± 0.1 °C. Carbonate-free KOH solutions of known concentrations (ca. 0.2 M) were used as the titrant. HCl stock solutions were prepared from concentrated HCl, and their concentrations were determined by potentiometric titrations using Gran’s method [22]. A Mettler Toledo T5 titrator equipped with a Mettler Toledo DGi 114-SC was used for the pH-metric measurements. The electrode system was calibrated according to Irving et al. [23]; the pH-metric readings could therefore be converted into hydrogen ion concentration. The water ionization constant, pK_w_, was 13.76 ± 0.01 under the conditions employed. Titrations were performed in the pH range 2.0—11.0. The initial volume of the samples was 15.00 mL, the metal concentrations were varied in the range of 1.0—5.0 mM. Samples with 1:1, 1:2 and 2:1 metal-ion-to-ligand ratios were titrated until reaching pH-equilibrium. Reproducibility of the equilibrium titration points included in the evaluation was within 0.005 pH units. The samples were in all cases completely deoxygenated by bubbling purified argon for ca. 15 min before the measurements. By using pH-potentiometric experimental data, overall stability constants (*β*_p,q,r_ = [M_p_H_q_L_r_]/[M]^p^[H]^q^[L]^r^) were calculated, where “M^2+^” stands for a half-sandwich type cation, [(η^6^-*p*-cym)Ru]^2+^ or [(η^5^-Cp*)Rh]^2+^, while “L” represents the deprotonated forms of the ligands. The PSEQUAD and SUPERQUAD computer programs were used to fit the titration curves [24,25]. During the calculations, protonation processes of the ligands and hydrolysis of the metal ions as concurring processes were taken into consideration. The hydrolytic constants involved in the equilibrium models were determined in previous works under the same conditions as used in this study (log*β*[{Ru(η^6^-*p*-cym)}_2_(μ^2^-OH)_2_]^2+^ = −7.12; log*β*[{Ru(η^6^-*p*-cym)}_2_(μ^2^-OH)_3_]^+^ = −11.88; log*β*[{Rh(η^5^-Cp*)}_2_(μ^2^-OH)_2_]^2+^ = −11.12; log*β*[{Rh(η^5^-Cp*)}_2_(μ^2^-OH)_3_]^+^ = −19.01) [26,27]. These values indicate that the hydrolysis of [(η^6^-*p*-cym)Ru]^2+^ starts above a pH of 3.5 while for [(η^5^-Cp*)Rh]^2+^ above a pH of 6.0.

^1^H NMR spectra were acquired on a Bruker Avance I 400 MHz NMR spectrometer in D_2_O at 25 °C in the presence of 0.20 M KNO_3_ or KCl and referenced to sodium 3-(trimethylsilyl)-1-propanesulfonate (TSP) or to the ^1^H resonances of the residual solvents. The measurements were carried out at a ca. 1:1 and 2:1 metal-ion-to-ligand ratio and c_L_ = 5 mM in order to register the pH dependence of the chemical shifts of the nuclei of the ligands. NaOD, DCl or DNO_3_ were used to set up the pH. Individual samples were equilibrated at least for 1 h before the measurements. pH* values (direct pH-meter readings in a D_2_O solution of a pH-meter calibrated in H_2_O according to Irving et al. [23]) were converted to pH values measurable at an ionic strength of 0.20 M using the following equation: pH = 0.930pH* + 0.40 [28].

ESI-TOF MS measurements in the positive mode were carried out on a Bruker maXis II UHR ESI-TOF MS instrument at the Department of Inorganic and Analytical Chemistry, University of Debrecen. For the mass spectrometric analysis of the solutions, the measurements were performed in water at a 0.03 mM metal ion concentration at different pH values using 1:1 and 2:1 metal-ion-to-ligand ratios. The instrument was equipped with an electrospray ion source, where the voltage was 4.5 kV. The drying gas was N_2_. The flow rate was 4 L/min, and the drying temperature was 200 °C. Na-formate was injected after each measurement, enabling internal *m/z* calibration. The spectra were evaluated with Bruker Compass Data Analysis 4.4. software.

### 2.4. X-ray Diffraction Analysis

**Figure 2:**
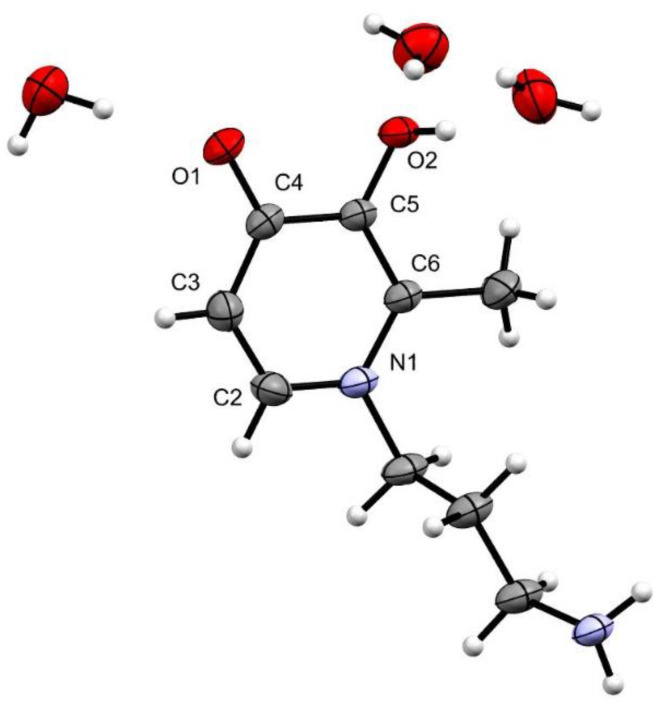
Molecular structure of H(L1) showing thermal displacement ellipsoids, drawn at the 50% probability level. Key bond lengths (Å) and angles (°) are C4-O1 1.289(3), C5-O2 1.315(3), C3-C2 1.372(3), C6-C5 1.392(3), C2-N1 1.341(3), C6-N1 1.372(3); O1-C4-C5 119.38(19), O2-C5-C4 119.66(18), C2-N1-C6 120.60(18).

**Figure 3:**
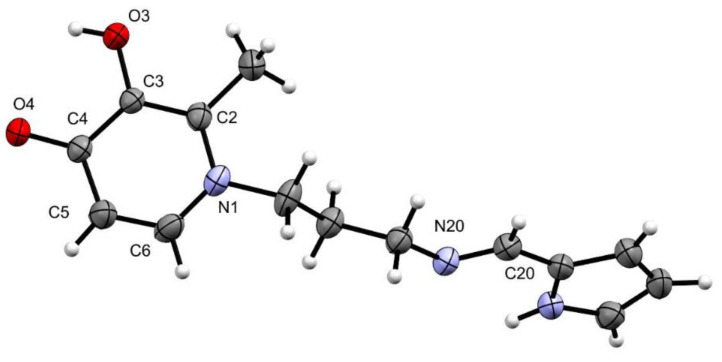
Molecular structure of H(L2) showing thermal displacement ellipsoids, drawn at the 50% probability level. Key bond lengths (Å) and angles (°) are C4-O4 1.269(2), C3-O3 1.354(2), C3-C2 1.371(3), C6-C5 1.351(3), C2-N1 1.369(3), C6-N1 1.351(3), N20-C20 1.265(3); O4-C4-C3 121.14(18), O3-C3-C4 119.94(17), C2-N1-C6 120.05(17).

X-ray quality crystals of H(L1) and H(L2) were grown by slow evaporation of the appropriate mother liquors. A suitable crystal was fixed under a microscope onto a Mitegen loop using high-density oil. Diffraction intensity data collection was carried out using a Bruker-D8 Venture diffractometer equipped with INCOATEC IμS 3.0 dual (Cu and Mo) sealed tube micro sources and Photon II Charge-Integrating Pixel Array detector using Mo Kα (λ = 0.71073 Å) radiation. The structures were determined using room temperature data collection. High multiplicity data collection and integration were performed using the APEX3 (Ver. 2017.3-0, Bruker AXS Inc., 2017) software. Data reduction and multi-scan absorption correction were performed using SAINT (Ver. 8.38A, Bruker AXS Inc., 2017). The structures could be solved using direct methods and refined on F2 using the SHELXL program [29] incorporated into the APEX3 suite. Refinement was performed anisotropically for all non-hydrogen atoms. Hydrogen atoms were placed into geometric positions, except for N–H and O–H protons that were located on the difference electron density map, and the N–H or O–H distances were constrained. The CIF file was merged and manually edited using the Publcif software [30]. Results of X-ray diffraction structure determinations were very good according to the Checkcif of PLATON software [31], and structural parameters, such as bond length and angle data, are in the expected range. The structures are also stabilized with weak C–H··O or C–H··N hydrogen bonds in several cases as well as strong hydrogen bonds with solvent water molecules. The crystallographic and refinement details can be seen in Table S1. Figure 2 and Figure 3 were made using the Mercury software. CCDC contains the supplementary crystallographic data for H(L1) and H(L2) with deposition numbers 2077921 and 2077922, respectively. These data can be obtained free of charge from The Cambridge Crystallographic Data Centre via www.ccdc.cam.ac.uk/data_request/cif (accessed on 7 June 2021).

## 3. Results and Discussion

### 3.1. Synthesis, Characterization and Acid-Base Properties of the Ligands

Synthesis of the novel ambidentate ligands was accomplished in a two-step process outlined in Scheme 1. H(L1) that was prepared according to a method in the literature [21] was first reacted with the appropriate aldehydes. The obtained Schiff-bases, H(L2) and H(L4), were then reduced with the aid of NaBH_4_ affording H(L3) and H(L5).

Both the intermediates and the desired final ligands were characterized using NMR, IR, ESI-TOF-MS and elemental analysis. All the analytical data (see Experimental Section) are consistent with the integrity and purity of the ligands. As a representative example, Figure S1 shows the NMR spectrum of the Schiff-base H(L4) and that of H(L5)·2 HCl after the NaBH_4_ reduction. The disappearance of the methine proton of H(L4) at 8.38 ppm and appearance of a singlet at 4.35 ppm for H(L5)·2 HCl unambiguously indicates the successful reduction and the formation of the secondary amine function in the chain. During the syntheses, we also managed to obtain single crystals of H(L1) and H(L2). X-ray diffraction analysis of them revealed the expected molecular structures that are shown in Figure 2 and Figure 3. The structural parameters such as bond length and angle data are in the expected range (for selected values, see also the captions of Figure 2 and Figure 3). Figure 2 indicates H(L1) in the neutral form with three water molecules co-present in the unit cell. Notably, the packing diagram of H(L1) in Figure S2 unravels the head-to-tail arrangement of the molecules showing consecutive layers of alkyamino chains and pyridinone moieties when seen in a view normal to the (100) plane stabilized by strong H-bonding. As can be seen in Figure 3, H(L2) also crystallized in the neutral form and, similarly to H(L1), its packing diagram (Figure S3) reflects the head-to-tail arrangement of the H(L2) molecules in the crystal with H-bonds between the pyridinone O atoms and H atoms attached to the pyrrole-N.

**Figure 4:**
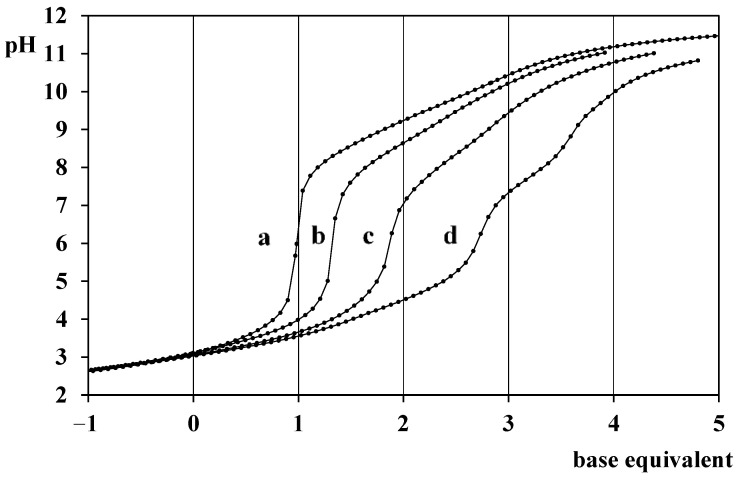
Representative titration curves of the H^+^–L3 (**a**) system and the [(η^5^-Cp*)Rh]^2+^–L3 system at 1:2 (**b**), 1:1 (**c**) and 2:1 (**d**) metal-ion-to-ligand-ratio s (c_L3_ = 2.45 mM). Negative base equivalent refers to an excess of acid in the sample.

**Figure 5:**
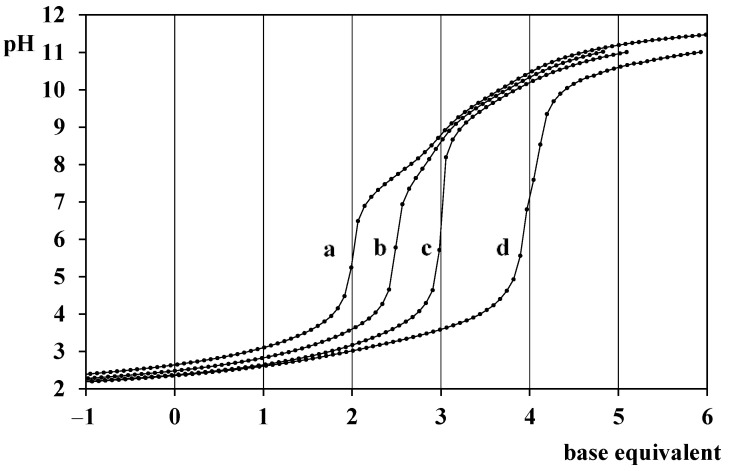
Representative titration curves of the H^+^–L5 (**a**) system and the [(η^5^-Cp*)Rh]^2+^–L5 system at 1:2 (**b**), 1:1 (**c**) and 2:1 (**d**) metal-ion-to-ligand-ratio s (c_L5_ = 2.49 mM). Negative base equivalent refers to an excess of acid in the sample.

To explore the acid-base properties of the novel, ambidentate ligands, H(L3) and H(L5), pH-potentiometry was used. Titration curves of the ligands in Figure 4 and Figure 5 (curves a) indicate one (H(L3)) and two (H(L5)) base consumption processes up to pH 4 and two further processes in the basic pH-range. The calculated dissociation constants appear in Table 1. Considering the structural similarities of H(L3) and H(L5) and the acid-base properties of Hdhp (Figure 1), also displayed in Table 1, as well as analysis of the pH-dependence of the chemical shifts of the corresponding NMR signals (Figures S4 and S5), the two high p*K* values of H(L3) and H(L5) should belong to the dissociation of the secondary ammonium and the OH group situated at the pyridinone ring. Although both of the small differences between these values for H(L3) and H(L5) and the NMR results support some overlapping proton dissociation processes from these sites, the highest p*K* values should belong to the pyridinone OH. Among the low p*K* values, 3.19 (H(L3)) and 3.20 (H(L5)) correspond to the deprotonation of the other OH group of the pyridinone ring. For H(L5), the low basicity of the pyridine N atom (p*K* = 5.2 for pyridine [32]) can be explained by the presence of an H-bond with the secondary ammonium group being in a chelatable position in line with the identical trend for pic (Table 1). Taking into consideration the slightly different experimental conditions, the tentative p*K* values (Table 1) obtained from the NMR data (Figures S4 and S5) agree reasonably well with those from the potentiometric titrations. Both the pH-metric and NMR results support the lack of deprotonation of the pyrrole-NH in the measurable pH-range. 

Representative pH-potentiometric titration curves are seen in Figure 4 and Figure 5, as well as in S6 and S7 for the [(η^5^-Cp*)Rh]^2+^–L3, [(η^5^-Cp*)Rh]^2+^–L5, [η^6^-*p*-cym)Ru]^2+^–L3 and [η^6^-*p*-cym)Ru]^2+^–L5 systems, respectively. The equilibrium models and overall stability constants, providing the best fit of the curves together with some derived constants are summarized in Table 1. For Pd(II), precipitation occurred with both of the ligands regardless of the metal-ion-to-ligand ratios from the beginning of the titrations; therefore, these solution equilibrium studies were discontinued, and thus, no data are shown in Table 1 for the Pd(II) systems.

Since the chelating functions of the ligands in this study (and in the models, Table 1) are protonated up to high pH (furthermore, the pyrrole-NH does not release its proton within the measurable pH-range), the metal ions must compete with protons for the binding sites. Moreover, hydrolysis of [η^6^-*p*-cym)Ru(H_2_O)_3_]^2+^ starts above pH ca. 3.5, while for [(η^5^-Cp*)Rh(H_2_O)_3_]^2+^ at pH ~ 6 [27]. In such cases, the overall stability constants (log*β* values) alone do not correctly measure the effectiveness of a ligand to bind a certain metal ion under a given condition; the effects of the overlapping protonation and hydrolytic processes need to be taken into account. This was done by calculating the conditional pM’ values (by definition, pM’ = −log[M]’ where [M]’ = the total concentration of the non-chelated metal ion) as usual, at c_M_ = 1μM, the metal-ion-to-ligand ratio = 1:10, abbreviated as pM’_1:10_, and shown in Table 1. However, because the equimolar condition (1:1 ratio of the components) is much more adequate in this study, the pM’ values were also calculated at c_M_ = c_L_ = 1 μM and are in Table 1 as pM’_1:1_ values. In both cases, a higher numerical value of pM’ (lower concentration of the non-chelated metal ion) indicates higher effectivity of the ligand to bind the metal ion [36].

Because H(L3) and H(L5) have two separated chelating moieties, the formation of different binding isomers of the complexes is possible. To obtain a somewhat deeper insight from solution equilibrium results into the binding modes, stability trends of the various chelates can be evaluated by using model ligands. 1,2-dimethyl-3-hydroxy-4(1*H*)-pyridinone (Hdhp, Figure 1) was used to model the (O,O) chelation with the two half-sandwich cations, while 2-picolylamine (pic, Figure 1) served as a model for the (N,N) chelate formed with the Rh-containing cation by the pyridine-N and the secondary amino-N of L5. (Unfortunately, neither the stability constant for the [η^6^-*p*-cym)Ru]^2+^ –pic system nor an adequate model for the (N,N)-chelating set of L3 are available).

To evaluate the effectiveness of the model mono-chelators in binding one of the studied cations at various pH-values when the above competing processes are also considered, conditional stability constants (logK’) were calculated as follows:

K’ = [ML]’_total_/[M]’_total_·[L]’_total_

where [ML]’_total_ = total concentration of the complexed half-sandwich cation; [M]’_total_ = total concentration of the metal cation in any form not complexed by the ligand; [L]’_total_ = total concentration of the ligand non-bonded to the metal ion.

**Figure 6:**
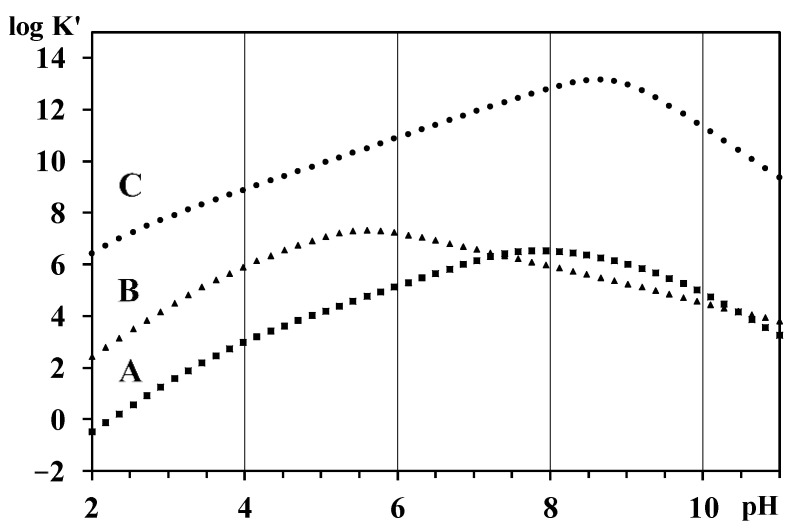
pH dependence of the calculated conditional stability constants (logK’) in the [(η^5^-Cp*)Rh]^2+^–dhp (**A**), [η^6^-*p*-cym)Ru]^2+^–dhp (**B**) and [(η^5^-Cp*)Rh]^2+^–pic (**C**) systems (t = 25 °C, I= 0.20 M, c_L_ = c_M_ = 5.0 mM) using data from References [33,34,35].

The calculated logK’ values as a function of pH are shown in Figure 6.

As Figure 6 shows, within the evaluated pH-range, the logK’ values are significantly lower compared to the corresponding overall log*β*_ML_ values (Table 1), indicating strong competition between the protonation/hydrolytic processes and the complexation in all the three model systems. The (N,N)-chelated [ML] complex in the [(η^5^-Cp*)Rh]^2+^–pic system has higher stability compared to the corresponding (O,O)-chelated one during the whole evaluated pH-range. However, the significantly higher effectivity of the (O,O)-chelate to bind the half-sandwich Ru-containing cation compared to the Rh-containing one exists only in the acidic pH range. Due to the higher affinity of [η^6^-*p*-cym)Ru]^2+^ towards hydrolysis, the difference between the two conditional stability constants starts decreasing above pH of ca. 5.5 and disappears by a ca. pH of 7 (cf. curves A and B in Figure 6). This information of the three model systems could be used in the evaluation of the results obtained with the novel bis-chelator ligands, H(L3) and H(L5).

### 3.2. Complexation Properties of H(L3)

Due to the separated (N,N) and (O,O) chelating sets within H(L3) titrations at 2:1 (in addition to 1:1 and 1:2) metal-ion-to-ligand ratios were also carried out. Titration curves with [η^6^-*p*-cym)Ru]^2+^ are shown in Figure S6, while those with [(η^5^-Cp*)Rh]^2+^ can be seen in Figure 4. Models providing the best fit of the experimental curves are summarized in Table 1. The models in Table 1 consist of differently protonated 1:1 complexes, while for [(η^5^-Cp*)Rh]^2+^, the assumption of various 2:1 species also significantly improved the fit of the titration curves. 

**Figure 7:**
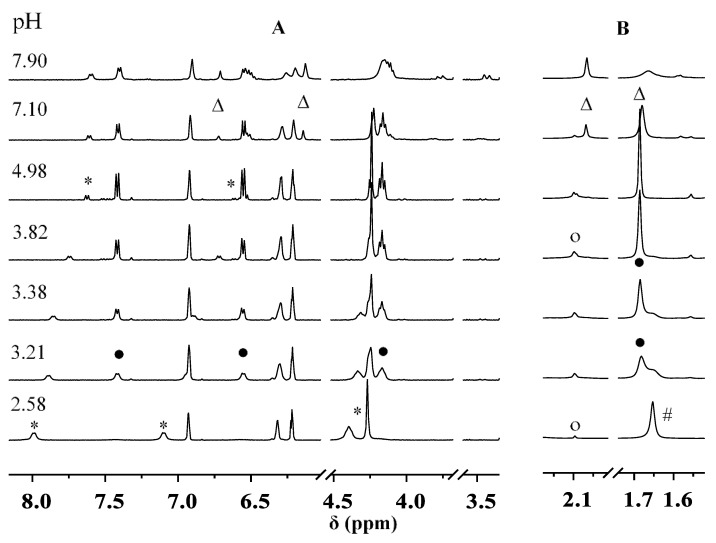
pH-dependence of the signals belonging to the L3 protons (**A**) and Cp* CH_3_ protons (**B**) of a 0.95:1 [(η^5^-Cp*)Rh]^2+^–L3 sample, c_L3_ = 5.0 mM. Symbols represent the signals belonging to the following species: H_x_(L3) (*), (O,O) coordinated [MHL]^2+^ (•), (N,N) coordinated [MH_2_L]^3+^ or [MHL]^2+^ (o), [M]^2+^ (#), [M_2_H_–1_L]^2+^ (Δ). The intensity scales are different for Figures. **A** and **B** for clarity.

**Figure 8:**
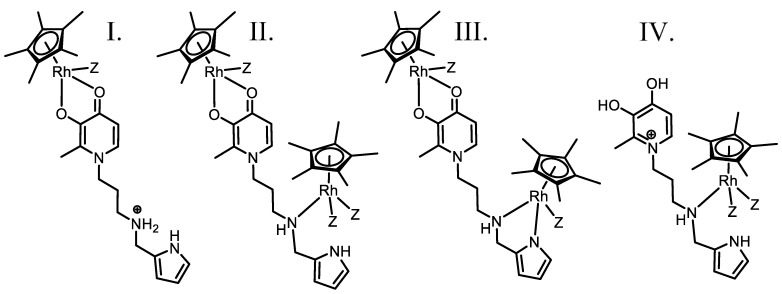
Suggested structures of the species in the [(η^5^-Cp*)Rh]^2+^–L3 system (Z = Cl^−^/H_2_O), charges of the metal ions are omitted for clarity. (**I**) [MHL]^2+^ (•); (**II**) [M_2_L]^3+^ (~); (**III**) [M_2_H_–1_L]^2+^ (η); (**IV**) [MH_2_L]^3+^ (o); symbols represent the notation of the species in the NMR figures.

To shed light on the most likely binding modes in the various species detailed, NMR studies were also carried out. pH-dependence of the NMR spectra registered in the [(η^5^-Cp*)Rh]^2+^–L3 system at 0.95:1 ratio (Figure 7) shows that at a pH of 2.58, signals of the uncomplexed ligand (*) and that of the free metal ion (#) as major species are present. On increasing the pH, a new set of major signals (•) appear attributable to [MHL]^2+^. A large upfield shift of the pyridinone ring protons at 6.55 and 7.42 ppm and the unchanged singlet multiplicity of the signal at 4.24 ppm belonging to the CH_2_ group located between the pyrrole ring and the secondary ammonium group are both consistent with the (O,O) coordination of the ligand to [(η^5^-Cp*)Rh]^2+^ (Figure 8I). A comparison of the NMR spectra registered at an excess of metal ions (Figure S8) reveals that besides the (O,O) coordinated [MHL]^2+^ (•), a new set of signals (~) also appear in the range 3.4–4.1 ppm for which the signal belonging to the CH_2_ group located between the pyrrole ring and the secondary ammonium group is an AB quartet. This strongly supports the involvement of N in the coordination, resulting in the loss of equivalence of the CH_2_ protons. Since in this pH range [M_2_L]^3+^ is the major species at the 2:1 metal-ion-to-ligand ratio (Figure S8), monodentate coordination of the secondary amine is assumed of the second [(η^5^-Cp*)Rh]^2+^ unit (Figure 8II). This is further supported by the NMR spectra registered in the more basic pH range since a further set of signals belonging to a major dinuclear species (η) can unambiguously be attributed to [M_2_H_–1_L]^2+^ (Figure 8III). Parallel with the formation of [M_2_H_–1_L]^2+^ above a pH of 7.0, the equivalence of the CH_2_ protons is completely lost in accordance with (N,N) coordination of L3 to the second metal entity after the metal-ion-assisted deprotonation and coordination of the pyrrole ring. Careful analysis of the Cp* signals at a 2.3:1 ratio (Figure S9) provides support for but also complements the above. As it can be seen in Figure S9 at a pH of 2.39 besides the signal of the uncomplexed aqua ion at 1.66 pm (#), a new signal (o) at 2.09 ppm appears. On increasing the pH the, the (O,O) coordinated complex (•) resonates at 1.69 ppm and becomes one of the major species. In addition, the two major dinuclear species [M_2_L]^3+^ (~) and [M_2_H_–1_L]^2+^ (η) can be detected. The small signal (o) at 2.09 ppm in the spectrum at a pH of 2.39 and the shoulder beside the (~) signal at a pH of 3.41 may suggest that, parallel with major (O,O) coordination, minor monodentate N coordination of the ligand also results in the formation of [MH_2_L]^3+^ or [MHL]^2+^ depending upon the protonation degree of the non-coordinating pyridinone unit (Figure 8IV). The spectra registered at the 1:1 ratio (Figure 7) indicate that the above 2:1 type complexes are co-present here too but as minor species together with a small amount of ligand in the free form. Above a pH of 8.0, precipitation in the samples hindered further analysis.

In the above species, coordination of either an (O,O) or an (N,N) chelate or both to one or two half-sandwich metal ion(s) makes the [(η^5^-Cp*)Rh]^2+^ unit(s) become stereogenic center(s). Upon (N,N) coordination, the secondary N also becomes a stereogenic center and, as a result, diastereomers may be formed. Since for [M_2_H_–1_L]^2+^, one Cp* signal can be detected for each of the metal centers, this may indicate that either only one diastereomer is present or the shielding of the Cp* protons are not measurably different in them or the interconversion of the various isomers is fast on the NMR time scale.

**Figure 9:**
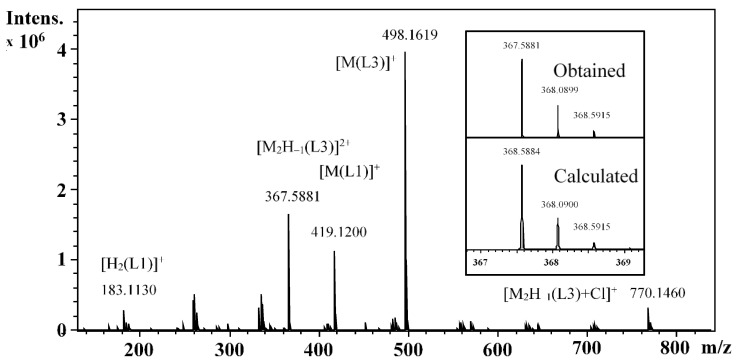
HR-ESI-TOF-MS spectrum registered in the [(η^5^-Cp*)Rh]^2+^–L3 system at 1:1 metal-ion-to-ligand ratio, pH = 7.0. Inlet shows the observed and calculated isotope pattern for [M_2_H_–1_L]^2+^.

High-resolution ESI-TOF-MS analysis provided further support for the NMR data and to the model from potentiometry. Table 2 summarizes the results indicating the presence of all the species discussed above. The measured and the calculated spectra showed the correct isotope pattern in all cases. As a representative example, the registered ESI-MS spectrum of the [(η^5^-Cp*)Rh]^2+^–L3 1:1 system at pH 7.0 is presented in Figure 9, while the inset shows the observed and calculated isotope pattern for [M_2_H_–1_L]^2+^.

For the [η^6^-*p*-cym)Ru]^2+^–L3 system, precipitation above pH 8.0 in the 2:1 sample allowed the evaluation of this latter curve in the acidic pH range only together with the others (Figure S6). Stability constants of the various 1:1 species (Table 1) are slightly higher than those for the organorhodium complexes, which is in agreement with previous literature data [33,34,35].

Comparison of the pH-dependent NMR spectra registered at 1:1 and 2:1 metal-ion-to-ligand ratio (Figures S10 and S11) reveals a similar complexation scheme in the acidic pH range as it was found with [(η^5^-Cp*)Rh]^2+^. The interaction with H(L3) starts with the formation of the (O,O) coordinated [MHL]^2+^, which is the major species (•) as low as the pH of the 2.1 at 1:1 ratio. In this sample, a small amount of uncomplexed metal ions (#) in the form of chlorido and aqua complexes and free ligand (*) can also be found; at a 2:1 ratio, the amount of the free organoruthenium cation increases. Below a pH of 7.0, the singlet multiplicity of the signal at 4.24 ppm belonging to the CH_2_ group situated between the pyrrole ring and the secondary ammonium group clearly indicates the absence of the N donor(s) from coordination. Precipitation in the NMR samples above pH 7.0 made the assignation of the signals more difficult, and unlike for the organorhodium system, at a 2:1 ratio, significant hydrolysis of the second, uncomplexed [η^6^-*p*-cym)Ru]^2+^ ion indicates the rather weak capability of the latter metal ion to promote the deprotonation and coordination of the pyrrole ring. The ESI-TOF-MS study of the various 1:1 and 2:1 metal-ion-to-ligand samples with lower concentrations (see Experimental Section) revealed the formation of [ML]^+^ but, in agreement with the NMR results, no 2:1 type complexes could be detected in a measurable amount (Table 2). Instead, significant hydrolysis of the organoruthenium cation resulting in the formation of [{(η^6^-*p*-cym)Ru}_2_(μ^2^-OH)_3_]^+^ (*m/z* = 522.0376) and [{(η^6^-*p*-cym)Ru}_2_(μ^2^-OH)(μ^2^-O)]^+^ (*m/z* = 504.0270) [37] was found. Notably, in these systems, fragmentation of the H(L3) ligand yielding H(L1) and its various organoruthenium and organorhodium complexes were also revealed under ESI-MS conditions.

Despite our efforts, we could not obtain single crystals from the solid precipitated out during the potentiometric and NMR studies. However, the fact that at a 2:1 metal-to-ligand-ratio, due to the hydrolysis of the excess metal ion, a large amount of [{(η^6^-*p*-cym)Ru}_2_(μ^2^-OH)_3_]^+^ is formed and remained in solution strongly suggests the 1:1 stoichiometry of the solid. In particular, its formation pH range may suggest an (O,O) coordinated species with a deprotonated secondary amino group and a chloride ion at the third coordination site of the metal ion to obtain [MLCl] with zero net charge. NMR study of the isolated yellow solid from a more concentrated sample in *d*_6_-DMSO supported its 1:1 stoichiometry (Figure S12) and the presence of some impurity or degradation products too. Although elemental analysis did not provide satisfactory results either, in the ESI-MS spectrum of the precipitated compound, two major signals were detected that can unambiguously be assigned to [{(η^6^-*p*-cym)Ru}L3]^+^ and [{(η^6^-*p*-cym)Ru}L1]^+^; the latter being formed under MS conditions only, due to fragmentation of L(3) (Figure S13). The change of the absorption band, assigned to υ(C=O), also indicates (O,O) complexation (1598 cm^–1^ in the spectrum of the complex and 1633 cm^–1^ for the free H(L3)) while the υ(C–N) signals did not change significantly (1341 and 1338 cm^–1^, respectively). It should also be mentioned, however, that even minor species in a solution can solely be precipitated out if they have got the least solubility among the various solutes.

### 3.3. Complexation Properties of H(L5)

Titration curves registered in the system [(η^5^-Cp*)Rh]^2+^–L5 are shown in Figure 5 while those with [η^6^-*p*-cym)Ru]^2+^ can be seen in Figure S7. The model providing the best fit of the experimental curves for the former system is shown in Table 1. With both metal ions, the complexation starts well below a pH of ~2; moreover, with [(η^5^-Cp*)Rh]^2+^, the titration curves indicated practically complete complexation at pH = 2.0. Therefore, the stability constant of [MH_2_L]^3+^ could only be obtained from independent NMR measurements where the 0.20 M ionic strength was set up with the partial replacement of KCl by HCl (Figure S14). The log*β*_[MH2L]_ was calculated considering the ratio of the integral of the various signals belonging to the complexed and uncomplexed ligand at known c_M_, c_L_ and c_H_ in the range 1.0 < pH < 2.0. [MH_2_L]^3+^ was proven as an (N,N) coordinated species (vide infra), and this is further supported by the excellent agreement in the acidity of the non-coordinating pyridinone ring of the coordinated ligand (p*K*_[MH2L]_ = 3.13) and the corresponding dissociation constant of the free ligand (p*K*_OH_ = 3.20, see Table 1). Therefore, below a pH of 2.0, the existence of no other species besides [MH_2_L]^3+^ needs to be considered. The model for the organorhodium system in Table 1 consists of differently protonated 1:1 and various 2:1 complexes and was obtained by keeping the stability constant of [MH_2_L]^3+^ fixed during calculations. Unlike the organorhodium system, with [η^6^-*p*-cym)Ru]^2+^, very slow complexation processes were detected. In particular, within a reasonable time, practically steady pH-readings could be achieved (most probably due to the relatively fast formation of kinetic products) during the potentiometric titrations; however, NMR studies indicated spectral changes especially at the 2:1 ratio even in 2 days (*vide infra*). This did not allow the evaluation of the titration curves for the [η^6^-*p*-cym)Ru]^2+^–L5 system.

**Figure 10:**
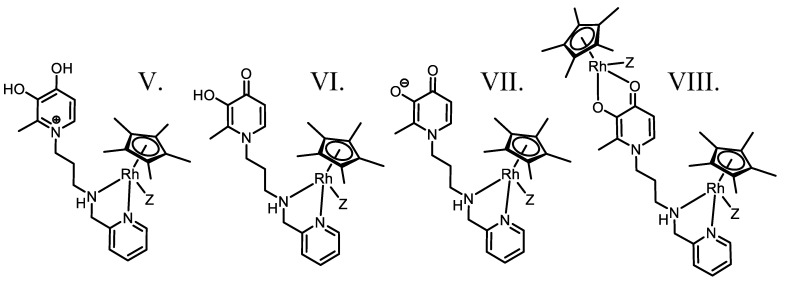
Suggested structures of the species in the [(η^5^-Cp*)Rh]^2+^–L5 system (Z = Cl^–^/H_2_O), charges of the metal ions are omitted for clarity. (**V**) [MH_2_L]^3+^ (•); (**VI**) [MHL]^2+^ (o); (**VII**) [ML]^+^ (▪); (**VIII**) [M_2_L]^3+^ (Δ); symbols represent the notation of the species in the NMR figures.

pH-dependence of the NMR spectra of the [(η^5^-Cp*)Rh]^2+^–L5 system at a 1:1 metal-ion-to-ligand ratio indicates significant complex formation well below pH = 2.0 (Figure S14). In the low field region in Figure S14, only a small upfield shift of the pyridinone signal (7.04 ppm) of the complex compared to that of the free ligand (7.19 ppm) accompanied by a much larger upfield shift of the signals of the pyridine protons (8.55 ppm for the complex vs. 8.90 ppm for the free ligand) together with the presence of the characteristic AB quartet belonging to the CH_2_ protons located between the pyridine ring and the secondary amino group of H(L5) in the range 4.0–4.5 ppm (Figure S15) are all consistent with the (N,N) coordination of the ligand. At pH ≤ 2.0, this complex is doubly protonated at the pyridinone OH groups (Figure 10V). On increasing the pH, a further upfield shift of the pyridinone ring protons in the range 2.0 < pH < 5.0 (Figure S15A) indicates the formation of [MHL]^2+^ (Figure 10VI) while above a pH of 8.0, the presence of [ML]^+^ (Figure 10VII) with stepwise deprotonation of the OH groups (what is also supported by the corresponding p*K*_MH2L_ and p*K*_MHL_ values, Table 1). The high-field region of the spectra (Figure S15B) shows two Cp* signals below pH 2.0, which are the only signals up to a pH of 8.1. The ratio of the integral values between the large and the smaller signal is constant in this whole pH range supporting the formation of two isomers. In fact, upon coordination of H(L5), both the metal ion and, due to the coordinating (N,N) chelating set, the secondary amino N become stereogenic centers, resulting in the formation of the two diastereomers.

**Figure 11:**
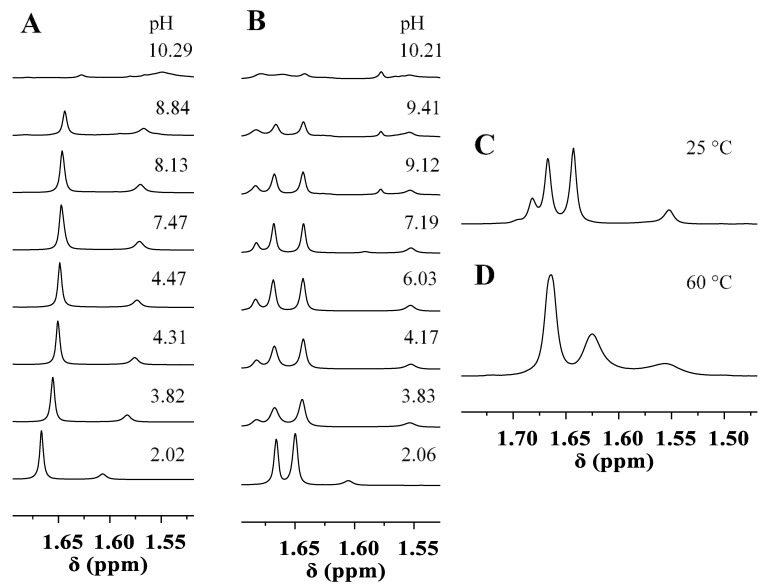
pH-dependence of the NMR signals belonging to the Cp* protons at 0.93:1 (**A**) and 2:1 (**B**) metal-ion-to-ligand-ratios in the [(η^5^-Cp*)Rh]^2+^–L5 system (c_L5_ = 5 mM). NMR signals of the Cp* protons at 2:1 ratio in the [(η^5^-Cp*)Rh]^2+^–L5 system at 25 °C (**C**) and 60 °C (**D**), pH = 4.35.

At a 2:1 metal-to-ligand-ratio (Figure S16) in the range 3.5 < pH < 9.4, a new set of signals (Δ) is indicative of the involvement of both the (O,O) and the (N,N) chelating sets yielding [M_2_L]^3+^ (Figure 10VIII). In particular, besides the presence of the AB quartet belonging to the CH_2_ protons as it was found at the 1:1 ratio, no continuous upfield shift of the ring protons of the pyridinone unit is detectable anymore. Instead, the resonances at 1.67 and 1.61 ppm, respectively, characteristic for [MH_2_L]^3+^ (•) (c.f. the spectra recorded at 1:1 ratio) show upfield shift upon the formation of [M_2_L]^3+^ (Δ). Above a pH of 9.0, the hydrolysis product of the metal ion, [{(η^5^-Cp*)Rh}_2_(μ^2^-OH)_3_]^+^ as a minor species at 1.58 ppm [27] also appears. A closer analysis of the signals belonging to the Cp* protons of the complexed metal ion (Figure 11) reveals that unlike the spectra at the 1:1 ratio (Figure 11A) in the 1.55–1.70 ppm range, two major and two minor signals are present in the range 3.8 < pH < 9.4 at a 2:1 metal-ion-to-ligand-ratio (Figure 11B and Figure S16). Since the sum of the integrals of the major and minor signals at 1.68 and 1.66 ppm are identical to those of the signals at 1.64 and 1.57 ppm, their appearance can be interpreted as the major and minor isomers of the (O,O) and the (N,N) coordinated [(η^5^-Cp*)Rh]^2+^ in [M_2_L]^3+^. This is supported by further NMR experiments at elevated temperature; as a representative example, Figure 11C,D shows the signals acquired at 25 and 60 °C. As it is seen due to the higher rate of the ligand exchange processes, the two signals at the lower field merged into one while for the two others, significant broadening can also be observed.

Since the (O,O) coordinated organorhodium complexes are typically more labile than those with (N,N) coordinating ligands [14], it is plausible to assume that the two peaks merging on heating (Figure 11D) originated from the (O,O) chelated metal ion while the two other belong to the two isomers of the (N,N) coordinated [(η^5^-Cp*)Rh]^2+^ entity. This latter finding is also in line with the observed chemical shift value range of the (N,N) coordinated species present as the single complex at the 1:1 ratio under acidic conditions (Figure S15).

**Figure 12:**
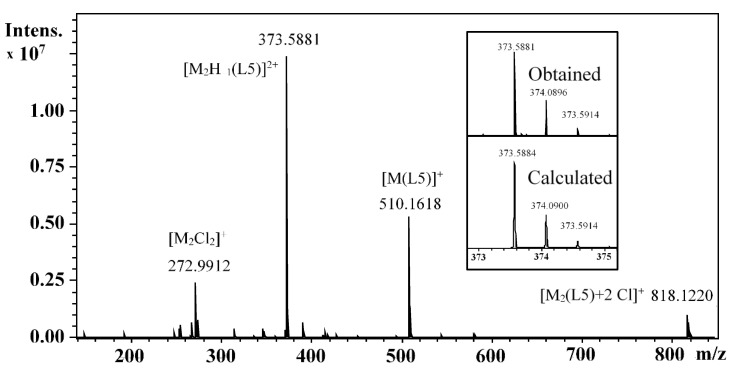
HR-ESI-TOF-MS spectrum registered in the [(η^5^-Cp*)Rh]^2+^–L5 system at 2:1 metal-ion-to-ligand-ratio, pH = 3.7. Inlet shows the observed and calculated isotope pattern for [M_2_H_–1_L]^2+^.

The MS study of the system at different ratios also provided proof for the presence of the various 1:1 and 2:1 species in solution (Table 2). As a representative example, the HR-ESI-TOF-MS spectrum registered for the 2:1 system at pH = 3.7 is presented while the inlet shows the obtained and calculated isotope pattern of [M_2_H_–1_L]^2+^ (Figure 12).

Similar to the organorhodium system, with [η^6^-*p*-cym)Ru]^2+^, the NMR data at the 1:1 ratio (Figure S17) supports the (N,N) coordination of the ligand under acidic conditions (see Figure 10V,VI, but with [η^6^-*p*-cym)Ru]^2+^). Although it is more difficult to see, this is indicated by the loss of equivalence of the CH_2_ protons situated between the two N donor atoms of L5. The assumed (N,N) coordination is clearly supported, however, either by the pyridinone signal exhibiting an upfield shift from 7.2 ppm (pH = 2.10) to 6.68–6.70 ppm by the pH = 7.0 characteristic for the deprotonation of the non-coordinated pyridinone ring or the downfield shift of the resonance of the pyridine proton at 8.70 ppm in the free ligand to 9.0 ppm in the complex at pH = 2.10. In addition, a minor species (Δ) corresponding to [M_2_L]^3+^ (vide infra) is also co-present in the fresh samples. Acquiring the spectra after 48 h (Figure S18), however, shows the disappearance of [M_2_L]^3+^, which strongly suggests that under these conditions it is a kinetic product. This finding supports the rather slow ligand exchange reactions of the (N,N) chelated complex in this system.

**Figure 13:**
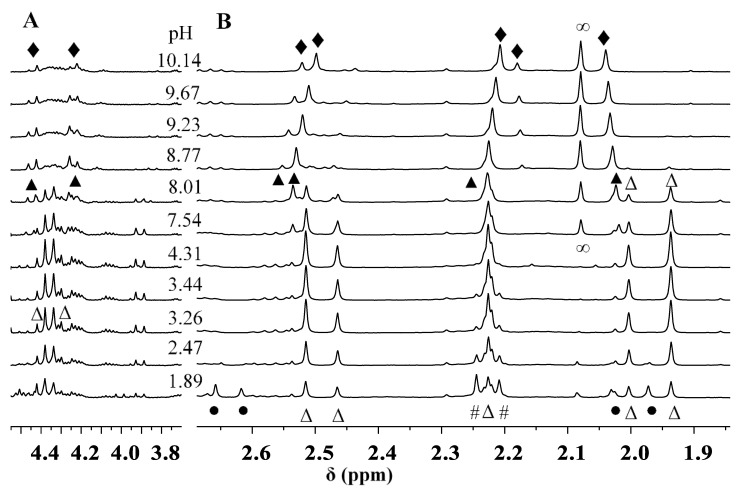
pH-dependence of the NMR signals detected in the [(η^6^-*p*-cym)Ru]^2+^–L5 system at 2.2:1 metal-ion-to-ligand-ratio, c_L5_ = 5.0 mM, t = 48 h. Symbols represent the signals belonging to the following species: [M(H_2_O)_3_]^2+^ and [M(Cl)_3_]^–^ (#), [MH_2_L]^3+^ (•), [M_2_L]^3+^ (Δ), [M_2_H_–1_L]^2+^ (▲), [M_2_H_–2_L]^+^ (♦) [M_2_(OH)_3_]^+^ (∞). The intensity scales are different for (**A**) and (**B**) for clarity.

At the 2:1 metal-ion-to-ligand-ratio, signals marked by “Δ” indicate a major species compared to the 1:1 samples supporting its dinuclear nature, the formation of an (O,O) and (N,N) chelated complex (Figure S19). In particular, looking at the 2.3–1.9 ppm range (signal of the p-cym Me group) (Figure 13) at pH 1.89 beside the uncomplexed metal ion ([(η^6^-*p*-cym)Ru(H_2_O)_3_]^2+^ at 2.24 ppm and [(η^6^-*p*-cym)Ru(Cl)_3_]^–^ at 2.21 ppm (#)), the (N,N) coordinated [MH_2_L]^3+^ (•) (as in Figure 10V but with [η^6^-*p*-cym)Ru]^2+^) and three new signals (Δ) most likely belonging to a [M_2_L]^3+^ type species (as in Figure 10VIII) can be seen. The presence of three major signals is consistent with the presence of diastereomers as both of the metal ions and the N atom of the secondary amino group become stereogenic centers upon formation of [M_2_L]^3+^ (Δ). Since [MH_2_L]^3+^ (•) contains an (N,N) coordinated ligand, the signal at 2.22 ppm should belong to the (O,O) coordinated ion while those at 2.00 and 1.94 ppm to the (N,N) coordinated metal ion. These latter two resonances can be rationalized by the two diastereomers discussed above also resulting in only one averaged signals for the (O,O) chelated metal ion in [M_2_L]^3+^ due to fast exchange reactions of the (O,O) chelator pyridinone unit on the NMR time scale. This is further supported by the NMR signals of the pyridinone ring (Figure 13, 2.45–2.68 ppm): for the ring methyl protons or ring hydrogens, two signals can be seen for each, a smaller and a larger one. The ratio of the appropriate signal pairs is identical to those belonging to the minor and major diastereomers discussed above. Above a pH of 8.0, the disappearance of [M_2_L]^3+^ (Δ) and the appearance of a species (▲) strongly suggest the formation of [M_2_H_–1_L]^2+^ (as in Figure 10VIII, but with one Z = OH^–^) upon deprotonation of one coordinating water in a slow process on the NMR time scale. A continuous shift of the signals of [M_2_H_–1_L]^2+^ (▲) above a pH 9.5 suggests a further deprotonation process yielding [M_2_H_–2_L]^+^ (♦) accompanied by a fast exchange on the NMR time scale. The fast nature and the pH range of this latter deprotonation process strongly suggests that it occurs at the water being coordinated together with the (O,O) chelate to one of the metal ions. This also means that the first deprotonation occurs at the (N,N) chelated organoruthenium cation in agreement with the formation of [M_2_H_–1_L]^2+^ (▲)in slow exchange with [M_2_L]^3+^ (Δ) and with the significant change of the shape of the AB quartet in the range 7.5 < pH <8.5. Due to the slight excess of Ru in the 2:1 sample, hydrolysis of this extra metal ion above pH 4 can also be detected. We have also found that on standing the amount of the [M_2_(OH)_3_]^+^ also increases supporting again the slow processes. Dependence of the spectrum (pH = 3.74) registered at the 2:1 ratio (Figure S20A) on increasing chloride ion concentration revealed the decrease of the number of signals (Figure S20B) attributable to various chloride/aqua coordinated dinuclear species due to displacement of the water molecules by Cl^–^ ions in the coordination sphere of [η^6^-*p*-cym)Ru]^2+^; however, these processes are also slow, as it is demonstrated in Figure S20C.

As in the case of the previous systems, the ESI-MS results fully support the presence of the major species in the [(η^6^-*p*-cym)Ru]^2+^ and L5 containing samples (Table 2).

## 4. Conclusions

Developing novel multitargeted metal complexes with high selectivity and lower side effects is an urgent need in the chemotherapy of cancers. Heterobimetallic Co(III)-PGM chaperon complexes with likely hypoxia activation can be remarkable candidates in this context. This type of compound requires rationally designed ligands with ambidentate, chelating donor atom sets for the two types of metal ions to bind. The new ligands presented in this study with separated (O,O) and (N,N) binding sets can be constituents of the above heterobimetallic complexes. To explore the fate of these types of complexes after administration and to obtain information on their stability and the pH-dependent binding capabilities of the organorhodium and –ruthenium entities with proven anticancer potential, these interactions were studied in solution with the combined aid of pH-potentiometry, NMR and HR ESI-MS.

Although a detailed kinetic study of the complex formation processes was beyond the scope of the present work, in agreement with previous results and based on indirect potentiometric and direct NMR evidences, organorhodium was found to form more labile complexes over ruthenium. In particular, [η^6^-*p*-cym)Ru]^2+^ forms more inert (N,N) coordinated complexes than Rh, while these processes are faster for (O,O) coordination with both metal ions. Furthermore, in agreement with data from the literature, related to half-sandwich type organoruthenium and –rhodium complexes with either chelating (N,N) or (O,O) donors, in most of the cases for the organoruthenium cation, the complex formation starts at lower pH (higher thermodynamic stability of the corresponding complexes) than for [(η^5^-Cp*)Rh]^2+^ but due to the higher affinity of [η^6^-*p*-cym)Ru]^2+^ towards hydrolysis, the complexed ligands are capable of competing with hydroxide ions to a lesser extent than for the Rh systems. As a result, under biologically relevant conditions, the Rh-binding effectivity of the ligands in this study (indicated, e.g., by the conditional stability of the complexes and by the corresponding pM’_1:1_ values) becomes comparable or even slightly higher than their effectivity towards Ru.

As far as the complexation capability of the two ligands is concerned, they differ in the presence of a pyrrole ring (L3) or a pyridine ring (L5) to be part of an (N,N) chelate with a secondary amino group. Since pyrrole is a much weaker acid (p*K*_a_ ~ 17 [38]) than the pyridinium cation (p*K*_a_ = 5.2) upon metal-ion-assisted deprotonation, the former is assumed to form a much stronger base and a (N,N) chelated complex with higher thermodynamic stability than for pyridine. However, due to the much higher basicity of the pyrrolate anion and the extent of the concurring process, its protonation becomes much more significant if the pH drops. Unfortunately, the model ligand for the (N,N)-chelate with pyrrolate was not found, but all the results with H(L3) and H(L5) support that, at least in the acidic pH range, the conditional stability constant of an M-(N,N) chelated species with pyrrolate involved is most probably much lower than that with pyridine-N; furthermore, it is even lower than the (O,O) one in the strongly acidic pH range. The trends obtained in this study provide support for those previously found regarding the pH-dependence of the (N,N) vs. (O,O) coordination of α-aminohydroxamates with these metal ions [13,14].

In the basic pH range, the proton competition decreases, but another concurring process, hydrolysis of the metal ion, becomes increasingly significant, resulting in a drop in the conditional stability constants with increasing pH.

Taken together, H(L3) is a less efficient (N,N) chelator for these metal ions than H(L5). Similarly, the relative effectivity of the (O,O) and (N,N) chelates (relative order of their conditional stability constants) determines that at a 1:1 metal-ion-to-ligand-ratio, H(L5) coordinates in a (N,N) manner to both cations in the whole pH range studied while for H(L3), the complexation starts with (O,O) coordination. At a 2:1 metal-ion-to-ligand-ratio, L3 cannot hinder the intensive hydrolysis of the second metal ion, although a small amount of 2:1 complex with [(η^5^-Cp*)Rh]^2+^ can also be detected.

Based on the results of this solution study, out of H(L5) and H(L3), the former one seems to be a better ligand to develop Co(III)/PGM heterodinuclear complexes involving (N,N)-chelated PGM and (O,O)-chelated Co(III) metal cores. Detailed solid-state studies are currently in progress in our laboratories.

## Figures and Tables

**Table 1:** Dissociation constants (p*K*) of the ligands, overall stability constants (log *β*), and some derived constants of their [(η^5^-Cp*)Rh]^2+^ and [η^6^-*p*-cym)Ru]^2+^ complexes at 25.0 °C and I = 0.20 M KCl ^a^.

	L3		L5		dhp		pic
p*K*_aromatic N_	–		1.3(1)	N/A ^c^	–		2.29 ^f^
p*K*_OH_	3.19(1)	3.46(2) ^c^	3.20(3)	3.50(4) ^c^	3.64 ^d^		–
p*K*_aliphatic N_	8.57(1)	8.53(7) ^c^	7.72(2)	7.73(8) ^c^	–		8.69 ^f^
p*K*_OH_	9.83(1)	9.61(5) ^c^	9.78(1)	9.60(9) ^c^	9.77 ^d^		–
	Rh^2+ g^	Ru^2+ g^	Rh^2+ g^		Rh^2+ g^	Ru^2+ g^	Rh^2+ g^
log*β*_[MH2L]_	–	–	23.7(3) ^c^		–	–	–
log*β*_[MHL]_	17.09(3)	20.30(6)	20.57(2)		–	–	–
log*β*_[ML]_	8.10(11)	11.92(4)	11.03(4)		8.93 ^d^	11.86 ^e^	13.59 ^f^
log*β*_[MH–1L]_	−3.1(3)	1.86(6)	0.00(5)		−2.97 ^d^	1.83 ^e^	–
log*β*_[M2L]_	14.98(4)	–	19.71(3)		–	–	–
log*β*_[M2H–1L]_	6.62(5)	–	9.30(6)		–	–	–
log*β*_[M2H–2L]_	–	–	−2.04(10)		–	–	–
Fitting parameter (mL) ^b^	0.0118	0.0115	0.0109		–	–	–
# of fitted data	455	303	289		–	–	–
p*K*_[MH2L]_	–	–	3.13		–	–	–
p*K*_[MHL]_	8.99	8.38	9.54		–	–	–
p*K*_[ML]_	11.20	10.06	11.03		11.90 ^d^	10.03 ^e^	8.48 ^f^
p*K*_[M2L]_	8.36	–	10.41		–	–	–
p*K*_[M2H–1L]_	–	–	11.34		–	–	–
pM’_1:10_^h^	7.08	9.76	11.26		7.53	10.04	13.23
pM’_1:1_^i^	6.27	6.73	8.48		6.39	6.83	9.15

^a^ 3σ standard deviations are in parentheses. ^b^ Fitting parameter is the average difference between the calculated and experimental titration curves expressed in mL of the titrant. ^c^ From ^1^H NMR; the value was kept fixed during the potentiometric calculations. ^d^ Taken from Reference [33]. ^e^ Taken from Reference [34]. ^f^ Determined using NMR data; taken from Reference [35]. ^g^ ”Rh^2+^” stands for [(η^5^-Cp*)Rh]^2+^ while ”Ru^2+^” for [η^6^-*p*-cym)Ru]^2+^. ^h^ pM’_1:10_ = −log[M]’, where [M]’ is the total equilibrium concentration of the metal ion in any form not complexed by the ligand, c_M_ = 1 µM; M:L = 1:10; t = 25 °C; I = 0.20 M, pH = 7.4. ^i^ pM’_1:1_ = −log[M]’, where [M]’ is the total equilibrium concentration of the metal ion in any form not complexed by the ligand, c_M_ = 1 µM; M:L = 1:1; t = 25 °C; I = 0.20 M, pH = 7.4. Calculations for the dhp and pic containing systems were based on data from References [33,34,35].

**Table 2:** Obtained and calculated *m/z* values for the various species in the studied systems.

				*m/z*	
Ligand	M^2+^	Species	pH (at 1:1/2:1 Ratios)	Obtained	Calculated
H(L3)	[(η^5^-Cp*)Rh]^2+^	[M(L3)]^+^	2.3; 5.1; 7.0; 9.1/2.3; 6.0; 8.6; 10.5	498.1619	498.1622
		[M(L1)]^+ a^	2.3; 5.1; 7.0; 9.1/2.3; 6.0; 8.6; 10.5	419.1200	419.1200
		[M_2_H_–1_(L3)]^2+^	5.1; 7.0; 9.1/2.3; 6.0; 8.6; 10.5	367.5881	367.5884
		[M_2_H_–1_(L3)+Cl]^+^	7.0; 9.1/6.0; 8.6; 10.5	770.1460	770.1461
		[H_2_(L1)]^+ a^	2.3; 5.1; 7.0; 9.1/2.3	183.1130	183.1128
		[M_2_Cl_2_]^+^	2.3/2.3, 6.0	272.9912	272.9912
		[M_2_(η-O)(η-OH)]^+^	/10.5	509.0430	509.0430
	[(η^6^-*p*-cym)Ru]^2+^	[M(L3)]^+^	2.3; 7.1/2.4; 4.0;	496.1537	496.1539
		[M(L1)]^+^	2.3; 7.1; 10.4/2.4; 4.0; 9.5;	417.1115	417.1116
		[H_2_(L1)]^+ a^	2.3; 7.1/2.4	183.1131	183.1128
		[M_2_(η-OH)_3_]^+^	10.4/9.5; 10.3	522.0374	522.0376
		[M_2_(η-O)(η-OH)]^+^	10.4/9.5; 10.3	504.0268	504.0270
H(L5)	[(η^5^-Cp*)Rh]^2+^	[M(L5)]^+^	2.2; 4.2; 8.5; 10.4/2.3; 3.7; 9.7; 10.3	510.1618	510.1622
		[MH(L5)+Cl]^+^	2.2; 4.2; 8.5; 10.4/2.3	546.1386	546.1389
		[MH(L5)]^2+^	2.2/2.3	255.5845	255.5848
		[M_2_Cl_2_]^+^	2.2; 4.2/2.3; 3.7; 9.7; 10.3	272.9912	272.9912
		[M_2_(L5)+2 Cl]^+^	4.2/2.3; 3.7; 9.7; 10.3	818.1220	818.1228
		[M_2_H_–1_(L5)]^2+^	4.2/2.3; 9.7; 10.3	373.5881	373.5884
	[(η^6^-*p*-cym)Ru]^2+^	[M(L5)]^+^	2.3; 7.9; 9.0/2.1; 4.3; 7.9; 10.0	508.1533	508.1539
		[MH(L5)+Cl]^+^	2.3; 7.9; 9.0/2.1; 7.9	544.1298	544.1303
		[M_2_H_–1_(L5)]^2+^	/2.1; 4.3; 7.9	371.0797	371.0806
		[MH(L5)]^2+^	2.3; 7.9/2.1; 4.3; 7.9	254.5795	254.5806
		[M(L5)+Cl]^+^	2.3	270.9814	270.9821
		[M_2_(η-OH)_3_]^+^	7.9; 9.0; 10.5/7.9; 10.0	522.0374	522.0376
		[M_2_(η-O)(η-OH)]^+^	7.9; 9.0; 10.5/7.9; 10.0	504.0268	504.0270
		[M_2_(L5)+2Cl]^+^	/2.1; 4.3;	814.1059	814.1058
		[M_2_(L5)+Cl]^2+^	/2.1; 4.3; 7.9	389.0681	389.0687

^a^ Under ESI-MS conditions, partial decomposition of H(L3) to H(L1) was also detected.

## Data Availability

The data presented in this study are available on request from the corresponding author.

## Supplementary Materials

The following are available online, Figures S1–S20: pH-potentiometric and NMR titration curves of the ligands and various systems, ESI-MS spectra, packing diagrams of H(L1) and H(L2), Table S1: Crystallographic parameters and refinement details for H(L1) and H(L2).
