# Supplementary material for: Donor Atom Preference of Organoruthenium and Organorhodium Cations on the Interaction with Novel Ambidentate (N,N) and (O,O) Chelating Ligands in Aqueous Solution
Source: Molecules. 2021 Jun 11;26(12):3586. doi: 10.3390/molecules26123586 (PMC8230953; doi:10.3390/molecules26123586)
Supplement: Supplementary file 1 [file molecules-26-03586-s001.zip › molecules-1213133-Supplementary.pdf]

Donor atom preference of organoruthenium and –rhodium cations on the interaction with novel ambidentate (N,N) and (O,O) chelating ligands in aqueous solution

Sándor Nagy, [a] András Ozsváth, [a] Attila Cs. Bényei, [b] Etelka Farkas, [a] Péter Buglyó [a,*]

[a] Department of Inorganic and Analytical Chemistry, University of Debrecen, H-4032 Debrecen, Egyetem tér 1, Hungary

[b] Department of Physical Chemistry, University of Debrecen, H-4032 Debrecen, Egyetem tér 1, Hungary

Supporting information

Table S1. Crystallographic parameters and refinement details for H(L1) and H(L2).

Chemical formula	$C_9H_{14}N_2O_2·3(H_2O)$ (H(L1))	$C_{14}H_{17}N_3O_2$ (H(L2))
M_r	236.27	259.30
Crystal system, space group	Triclinic, $P\bar{1}$	Monoclinic, $P2_1/c$
Temperature (K)	295	293
a, b, c (Å)	7.7713 (3), 8.3192 (4), 10.1279 (4)	9.4186 (8), 8.2937 (6), 17.7160 (14)
α, β, γ (°)	104.400 (2), 90.021 (2), 94.129 (2)	99.534 (3)
V (Å^3)	632.45 (5)	1364.77 (19)
Z	2	4
Radiation type	Mo $K\alpha$	
μ (mm^{-1})	0.10	0.09
Crystal size (mm)	0.75 × 0.39 × 0.14	0.19 × 0.18 × 0.15
Diffractometer	Bruker D8 VENTURE	
Absorption correction	Multi-scan *SADABS2016*/2 - Bruker AXS area detector scaling and absorption correction	
T_{min}, T_{max}	0.60, 0.99	0.87, 0.99
No. of measured, independent and observed [$I > 2\sigma(I)$] reflections	14222, 2492, 2036	12415, 2572, 1860
R_{int}	0.079	0.058
(sin θ/λ)$_{max}$ (Å^{-1})	0.618	0.609
$R[F^2 > 2\sigma(F^2)]$, $wR(F^2)$, S	0.076, 0.230, 1.07	0.049, 0.123, 1.05
No. of reflections	2492	2572
No. of parameters	173	175
No. of restraints	10	0
H-atom treatment		
	H atoms treated by a mixture of independent and constrained refinement	
(Δ/σ)$_{max}$	0.001	0.001
Δ〉$_{max}$, Δ〉$_{min}$ (e Å^{-3})	0.68, -0.31	0.19, -0.18

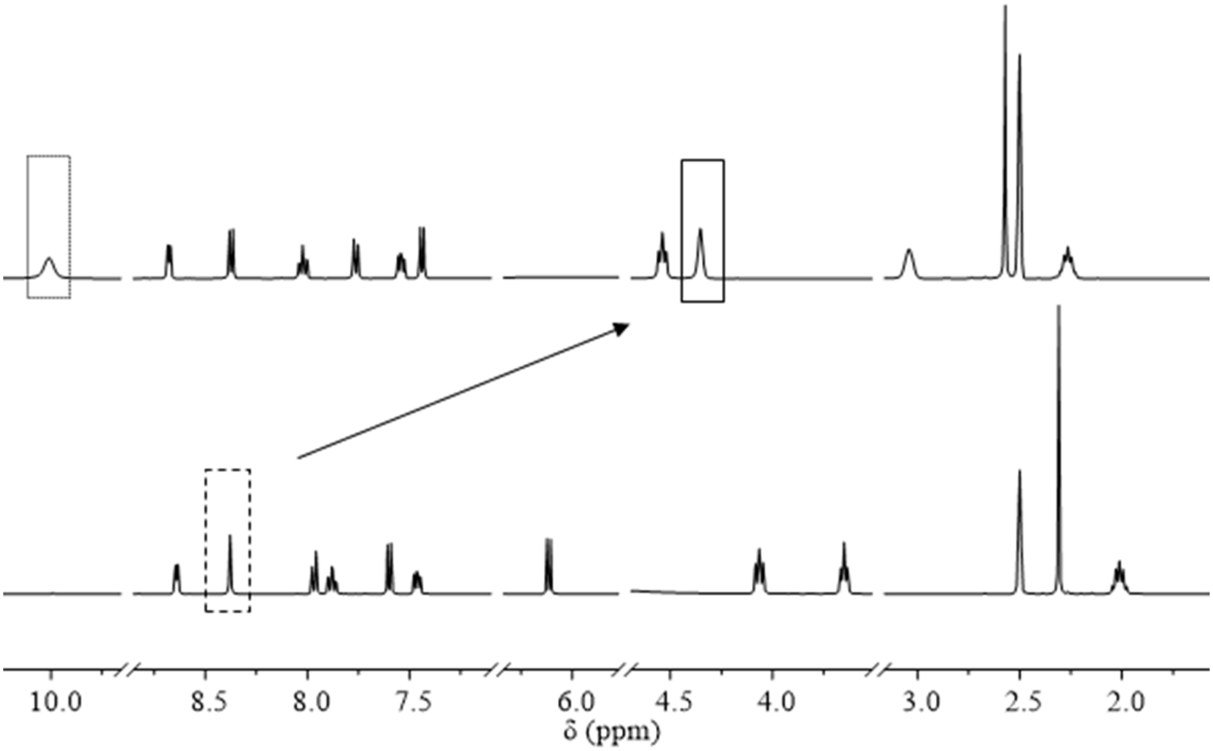

Figure S1. ^{1}H NMR spectrum of H(L4) (below) and H(L5)·2 HCl (above) in d^{6}-DMSO. The appearance of the signal belonging to the NH_2 porotons (10.01 ppm; dotted) in the aromatic and that of CH_2 (4.35 ppm; solid) in the aliphatic region of the H(L5)·2 HCl spectrum indicates a successful reduction.

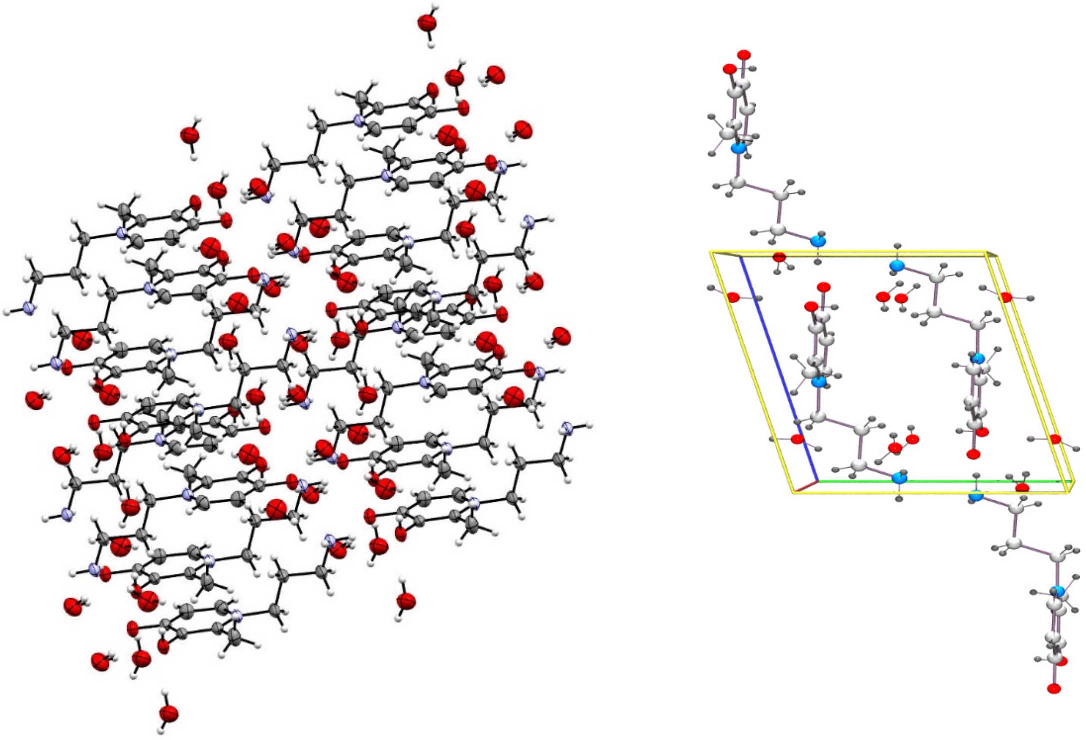

Figure S2. Packing diagram of H(L1) showing thermal displacement ellipsoids, drawn at the 50% probability level (left) and view normal to (100) plane (right).

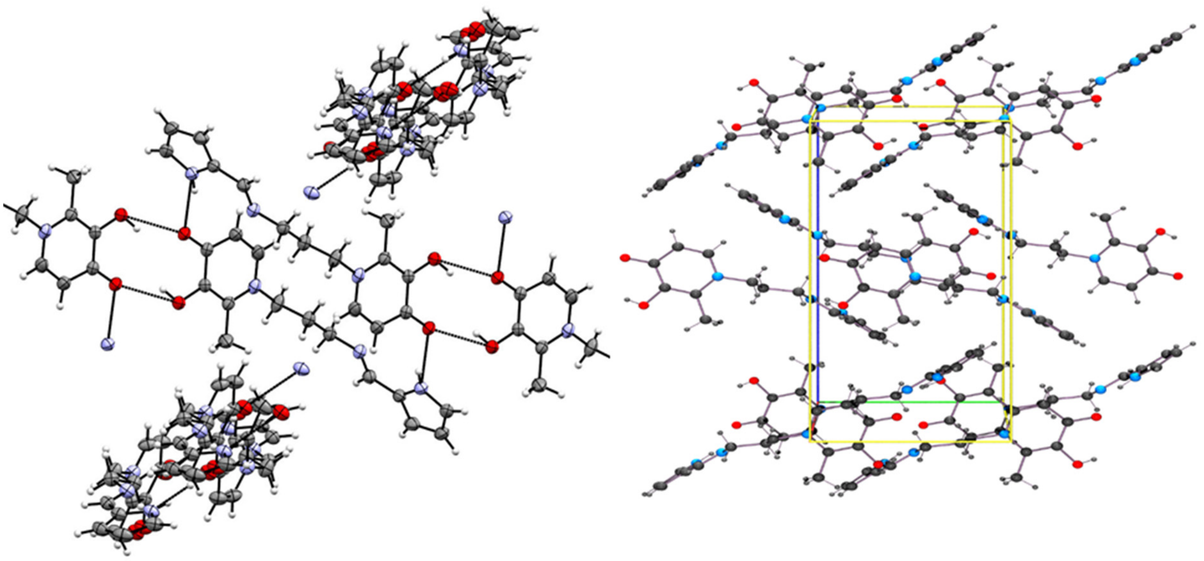

Figure S3. Packing diagram of H(L2) showing thermal displacement ellipsoids, drawn at the 50% probability level (left) and view normal to (100) plane (right).

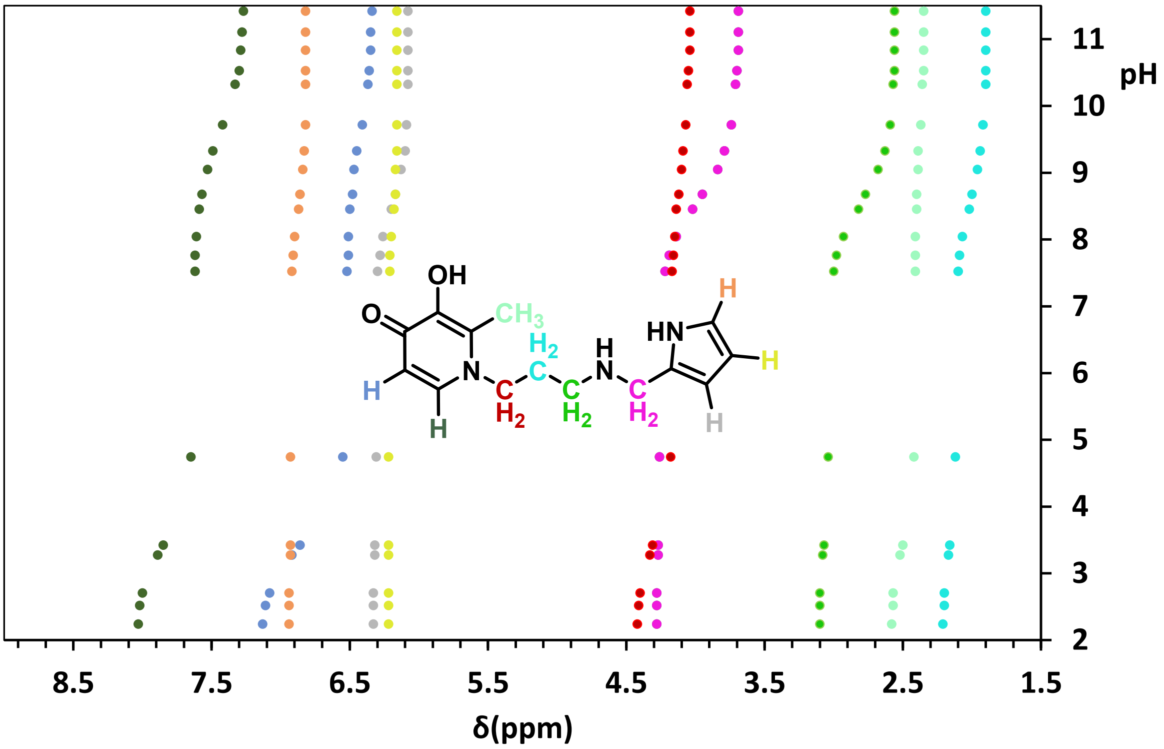

Figure S4. pH dependence of the chemical shifts of H(L3) in D_2O.

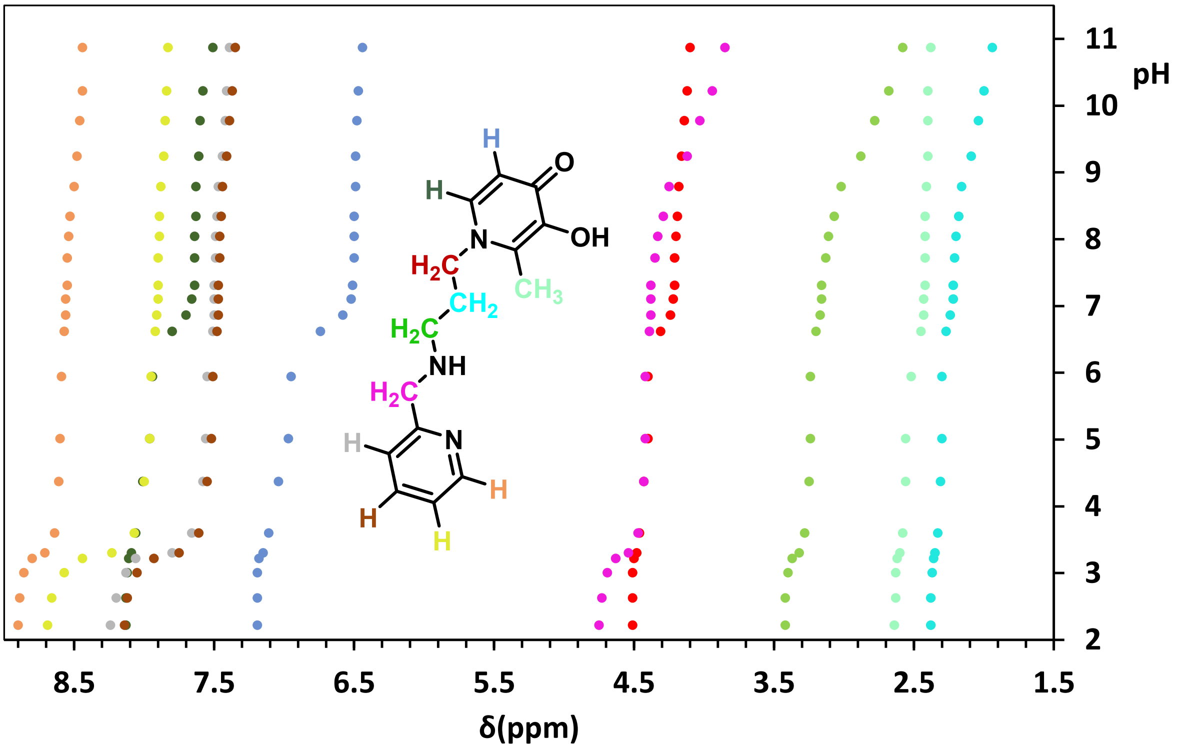

Figure S5. pH dependence of the chemical shifts of H(L5) in D_2O.

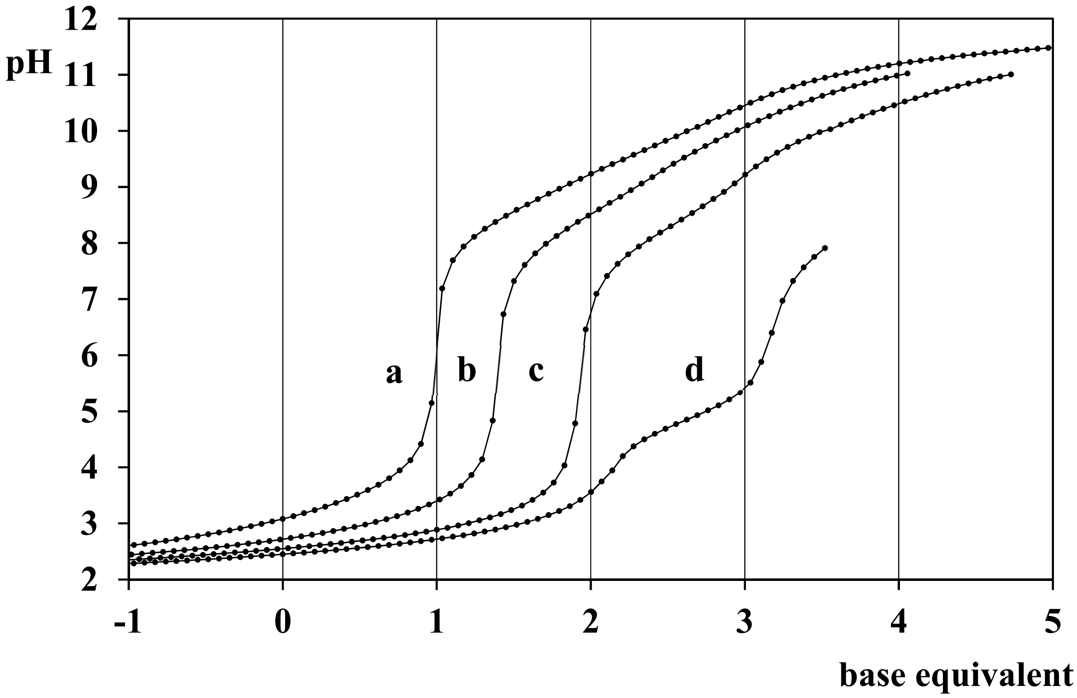

Figure S6. Representative titration curves of the H^+–L3 (a) system and the $[(\eta^6\text{-p-cym})Ru]^{2+}$–L3 system at 1:2 (b), 1:1 (c) and 2:1 (d) metal ion to ligand ratios (c_{L3} = 2.53 mM). Negative base equivalent refers to an excess of acid in the sample.

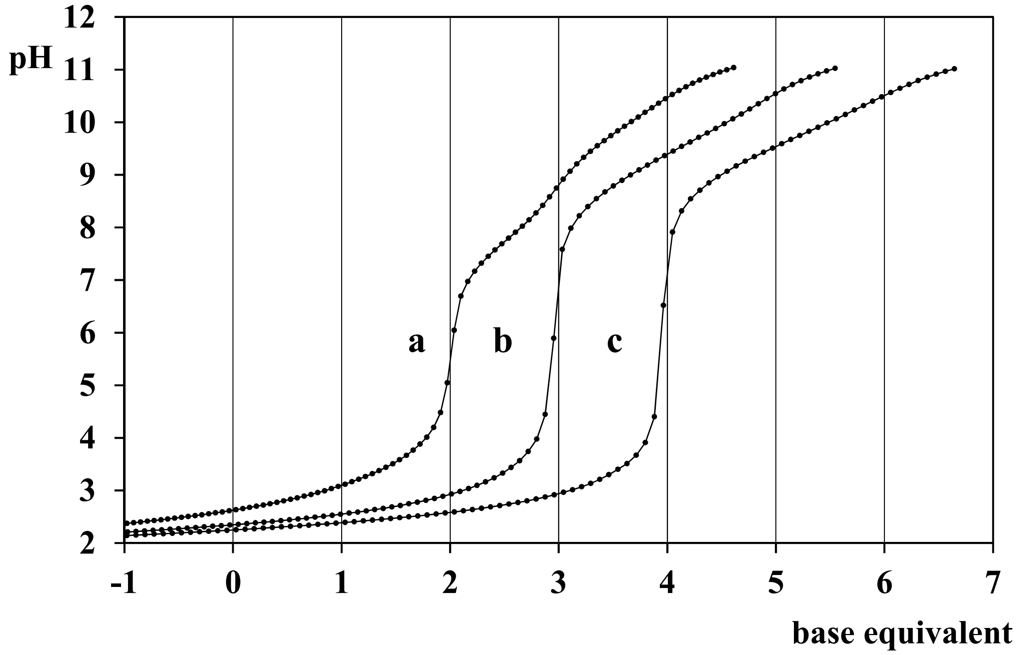

Figure S7. Representative titration curves of the H^+–L5 (a) system and the $[(\eta^6\text{-}p\text{-cym})Ru]^{2+}$–L5 system at 1:1 (b) and 2:1 (c) metal ion to ligand ratios (c_{L5} = 2.60 mM). Negative base equivalent refers to an excess of acid in the sample.

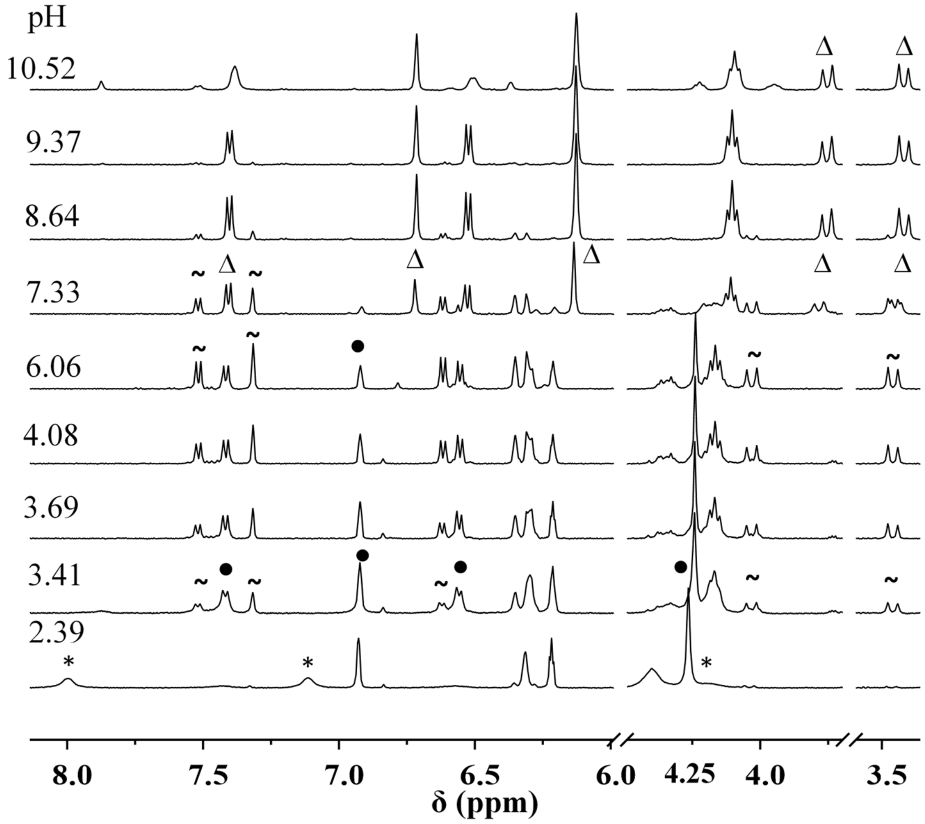

Figure S8. pH-dependence of the signals belonging to the L3 protons of a 2.3:1 $[(\eta^5\text{-}Cp^*)Rh]^{2+}$–L3 sample, c_{L3} = 5.0 mM. Symbols represent the signals of the following species: H_x(L3) (*), $[MHL]^{2+}$ (•), $[M_2L]^{3+}$ (~), $[M_2H_{-1}L]^{2+}$ (Δ).

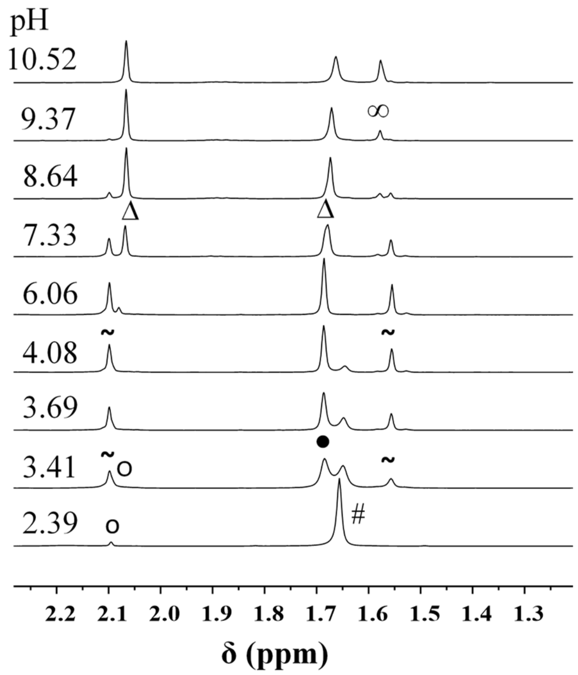

Figure S9. pH-dependence of the signals belonging to the Cp* CH_3 protons of a 2.3:1 $[(\eta^5$-Cp*)Rh$]^{2+}$–L3 sample, c_{L3} = 5.0 mM. Symbols represent the signals of the following species: H_x(L3) (*), $[(\eta^5$-Cp*)Rh$]^{2+}$ (#), (O,O) coordinated $[MHL]^{2+}$ (•), (N,N) coordinated $[MH_2L]^{3+}$ or $[MHL]^{2+}$ (o), $[M_2L]^{3+}$ (~), $[M_2H_{-1}L]^{2+}$ (Δ), $[M_2(OH)_3]^{+}$ (∞).

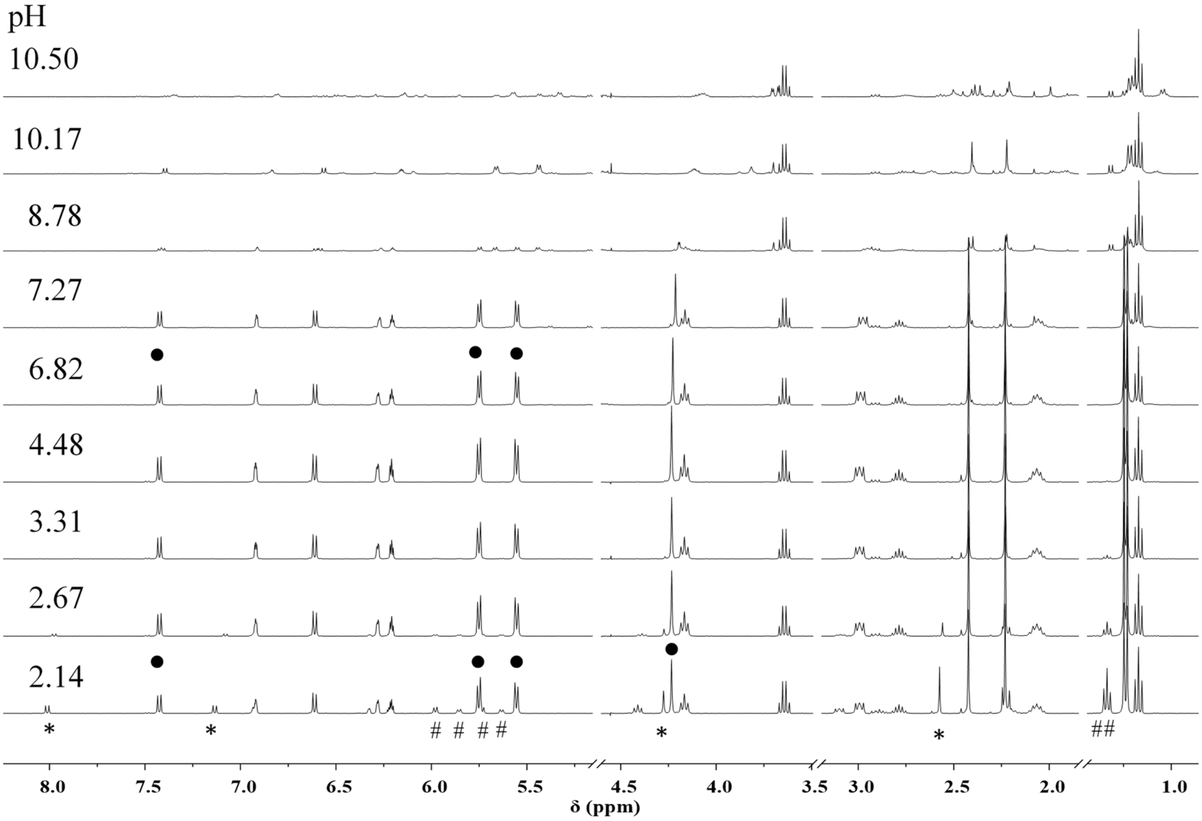

Figure S10. pH-dependence of the signals belonging to the protons in the $[(\eta^6\text{-}p\text{-cym})Ru]^{2+}$–L3 system at 1:1 ratio, c_{L3} = 5.0 mM. Symbols represent the signals of the following species: $H_x(L3)$ (*), $[M(H_2O)_3]^{2+}$ and $[M(Cl)_3]^-$ (#), $[MHL]^{2+}$ (•).

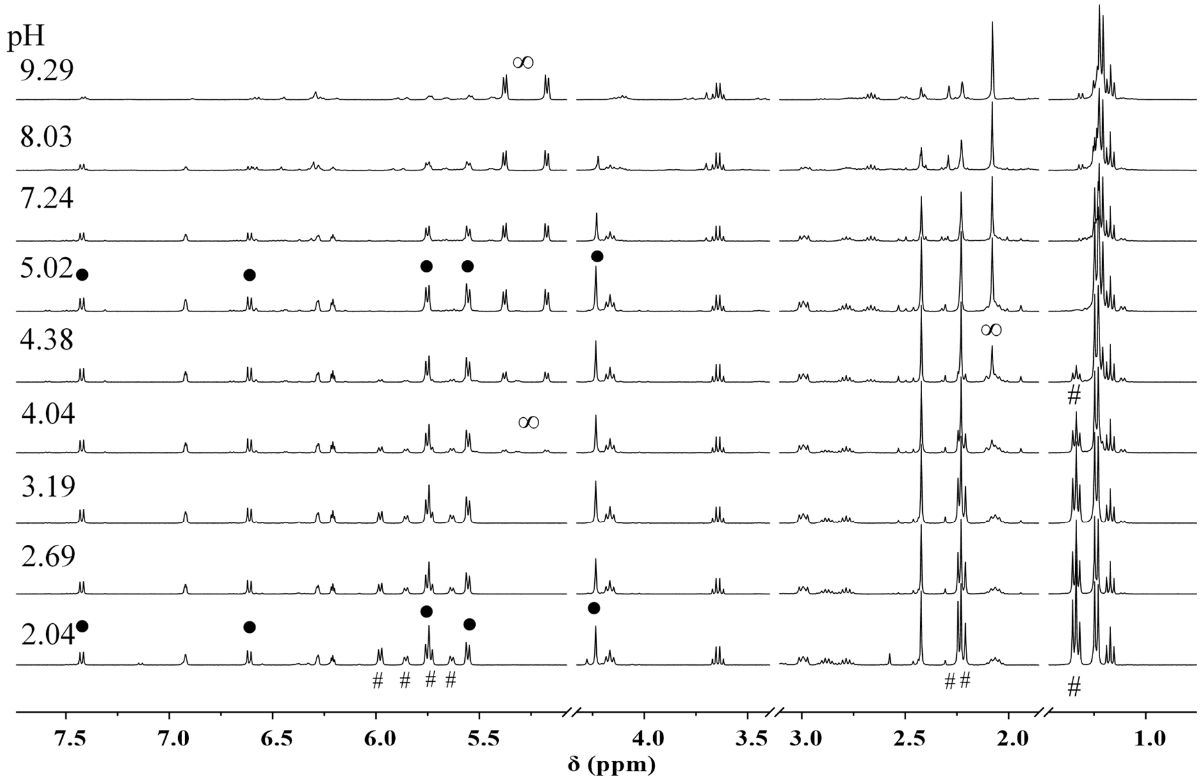

Figure S11. pH-dependence of the signals belonging to the protons in the $[(\eta^6\text{-}p\text{-cym})Ru]^{2+}$–L3 system at 2:1 ratio, c_{L3} = 5.0 mM. Symbols represent the signals of the following species: $H_x(L3)$ (*), $[M(H_2O)_3]^{2+}$ and $[M(Cl)_3]^-$ (#), $[MHL]^{2+}$ (•), $[M_2(OH)_3]^+$ (∞).

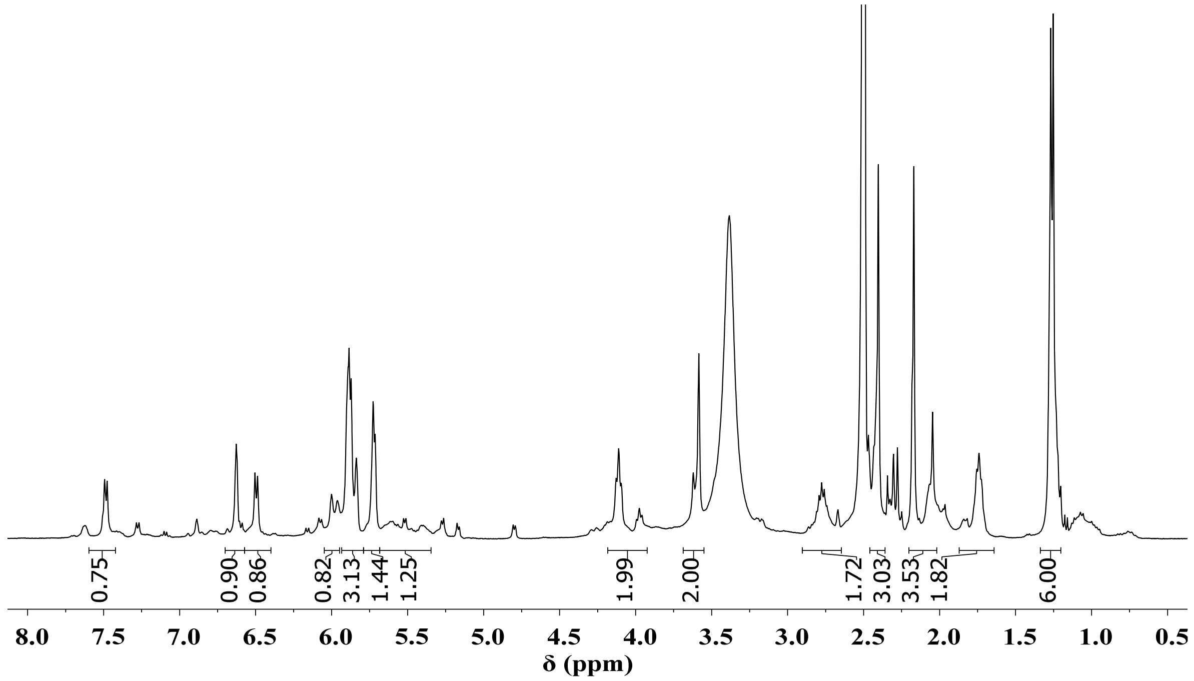

Figure S12. ^{1}H NMR spectrum of the precipitated yellow solid from a $[(\eta^6\text{-}p\text{-cym})Ru]^{2+}$–L3 1:1 sample, pH ~8, registered in d^6-DMSO.

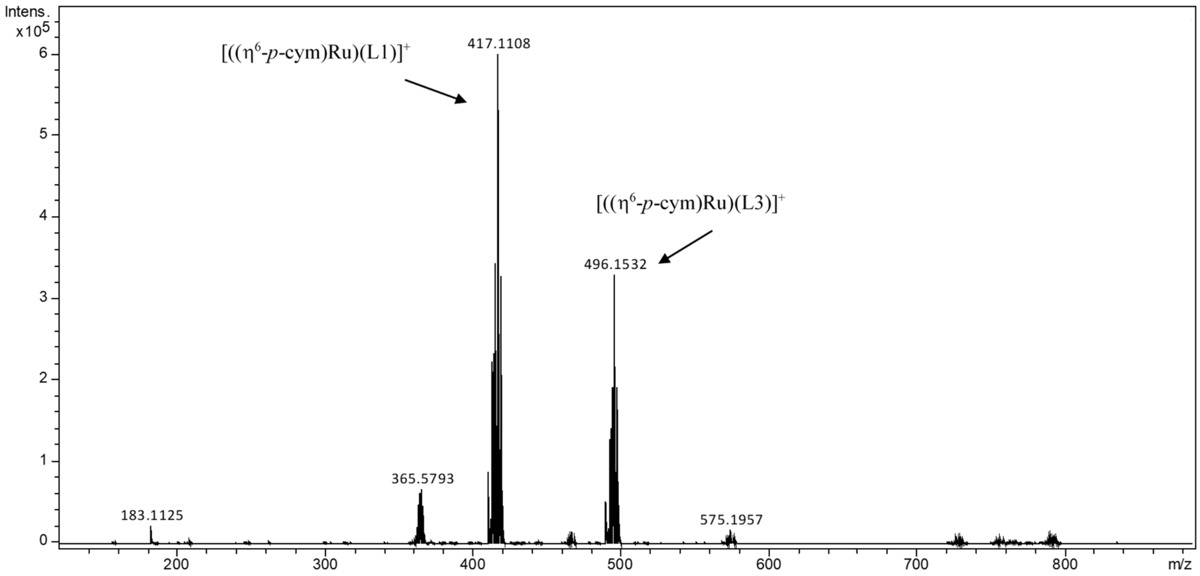

Figure S13. ESI-MS spectrum of the precipitated yellow solid from a [(η^6-*p*-cym)Ru]$^{2+}$–L3 1:1 sample.

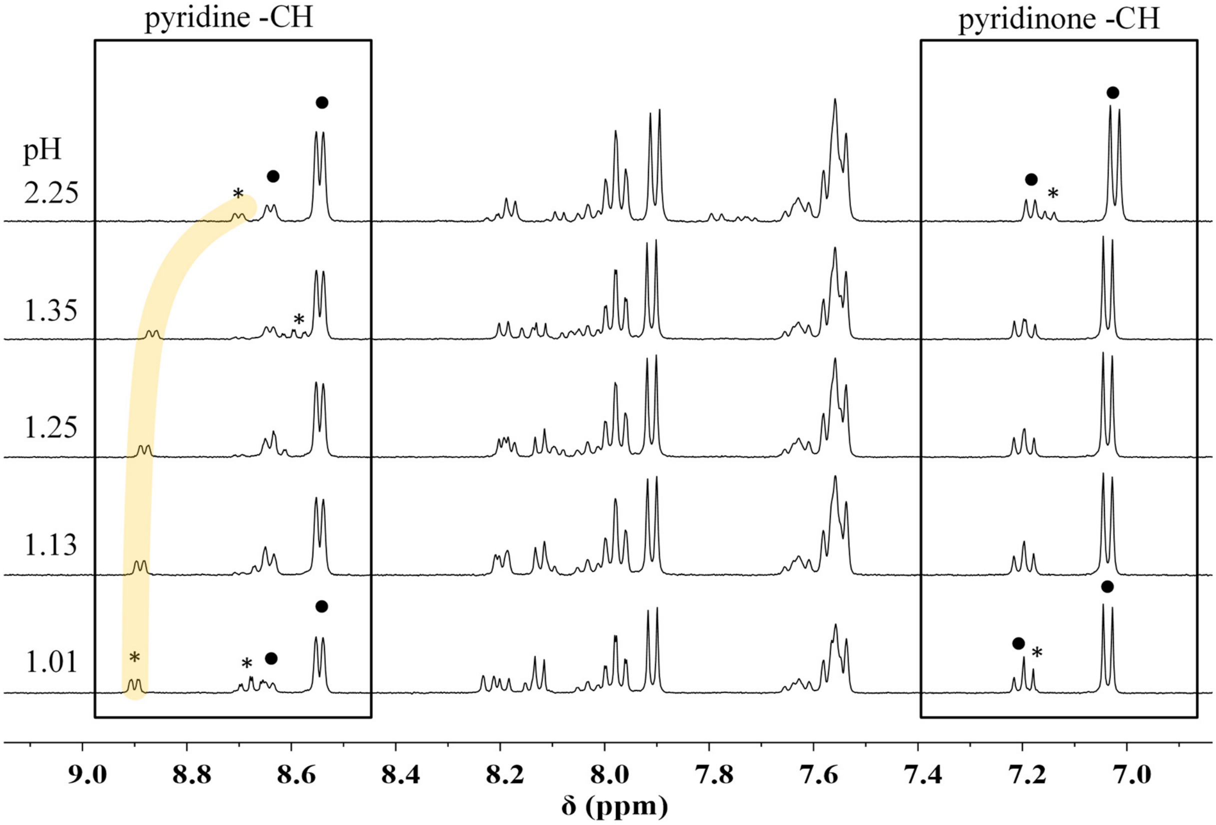

Figure S14. pH-dependence of the signals belonging to the L5 protons of a 1:1 $[(\eta^5\text{-Cp*})Rh]^{2+}$–L5 sample, c_{L5} = 5.0 mM. Symbols represent the signals of the following species: $H_x(L5)$ (*), $[MH_2L]^{3+}$ (•).

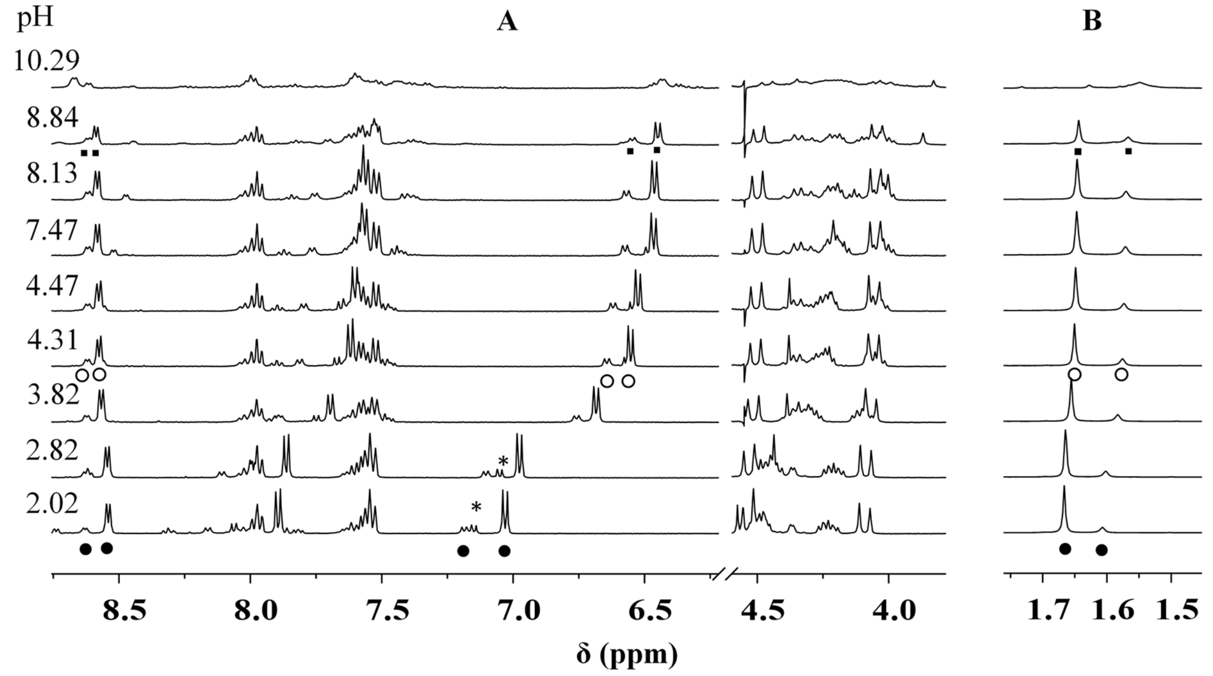

Figure S15. pH-dependence of the signals belonging to the L5 protons (A) and Cp* CH_3 protons (B) of a 0.93:1 $[(\eta^5\text{-Cp*})Rh]^{2+}$–L5 sample, c_{L5} = 5.0 mM. Symbols represent the signals of the following species: $H_x(L5)$ (*), $[MH_2L]^{3+}$ (•), $[MHL]^{2+}$ (○), $[ML]^{+}$ (▪).The intensity scales are different for Figs. A and B for clarity.

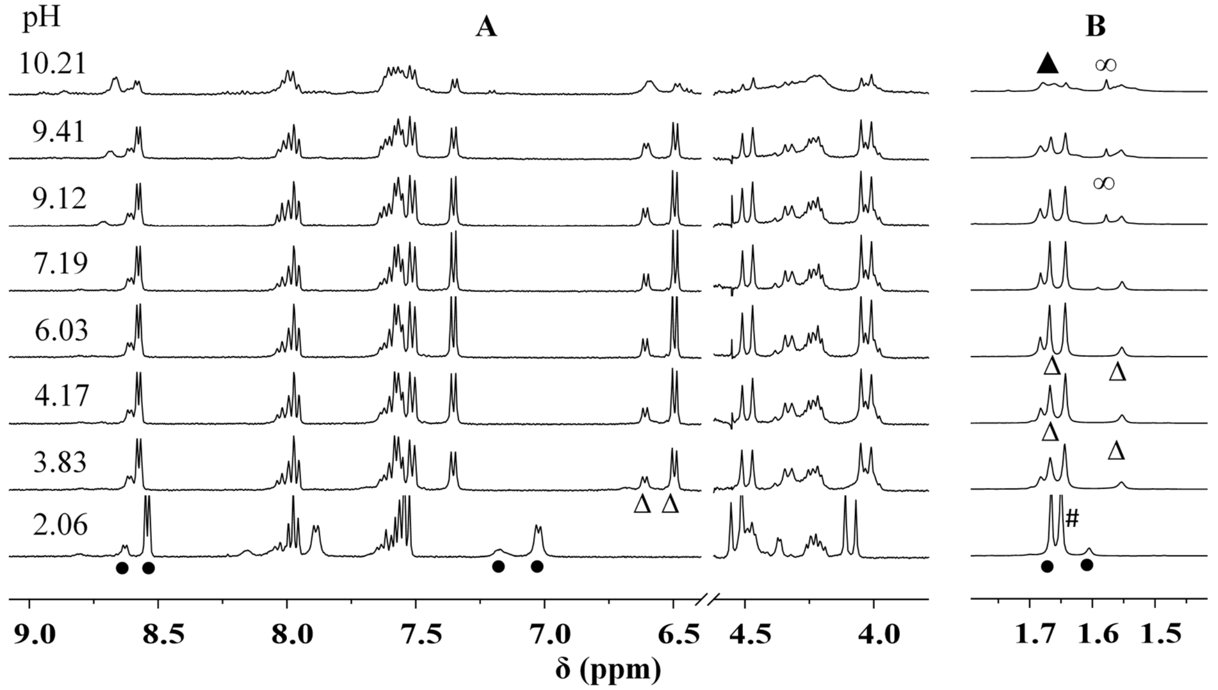

Figure S16. pH-dependence of the signals belonging to the L5 protons (A) and Cp* CH_3 protons (B) of a 2:1 $[(\eta^5\text{-Cp*})Rh]^{2+}$–L5 sample, c_{L5} = 5.0 mM. Symbols represent the signals of the following species: $[M(H_2O)_3]^{2+}$ and $[M(Cl)_3]^{-}$ (#), $[MH_2L]^{3+}$ (•), $[M_2L]^{3+}$ (Δ), $[M_2H_{-1}L]^{2+}$ (▲), $[M_2(OH)_3]^{+}$ (∞).

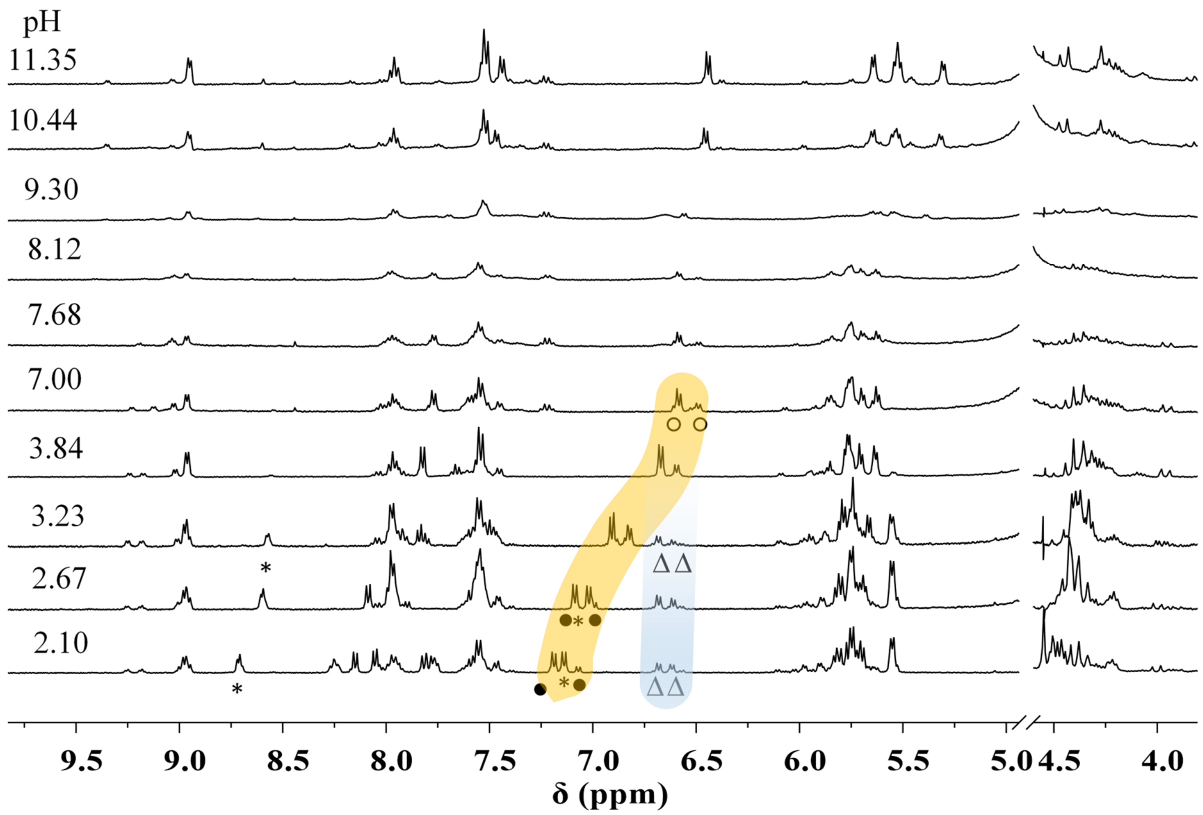

Figure S17. pH-dependence of the signals belonging to protons in the $[(\eta^6\text{-}p\text{-cym})Ru]^{2+}$–L5 system at 1:1 ratio, c_{L5} = 5.0 mM. Symbols represent the signals of the following species: $H_x(L5)$ (*), $[M(H_2O)_3]^{2+}$ and $[M(Cl)_3]^-$ (#), $[MH_2L]^{3+}$ (•), $[MHL]^{2+}$ (○), $[M_2L]^{3+}$ (Δ), $[M_2(OH)_3]^+$ (∞).

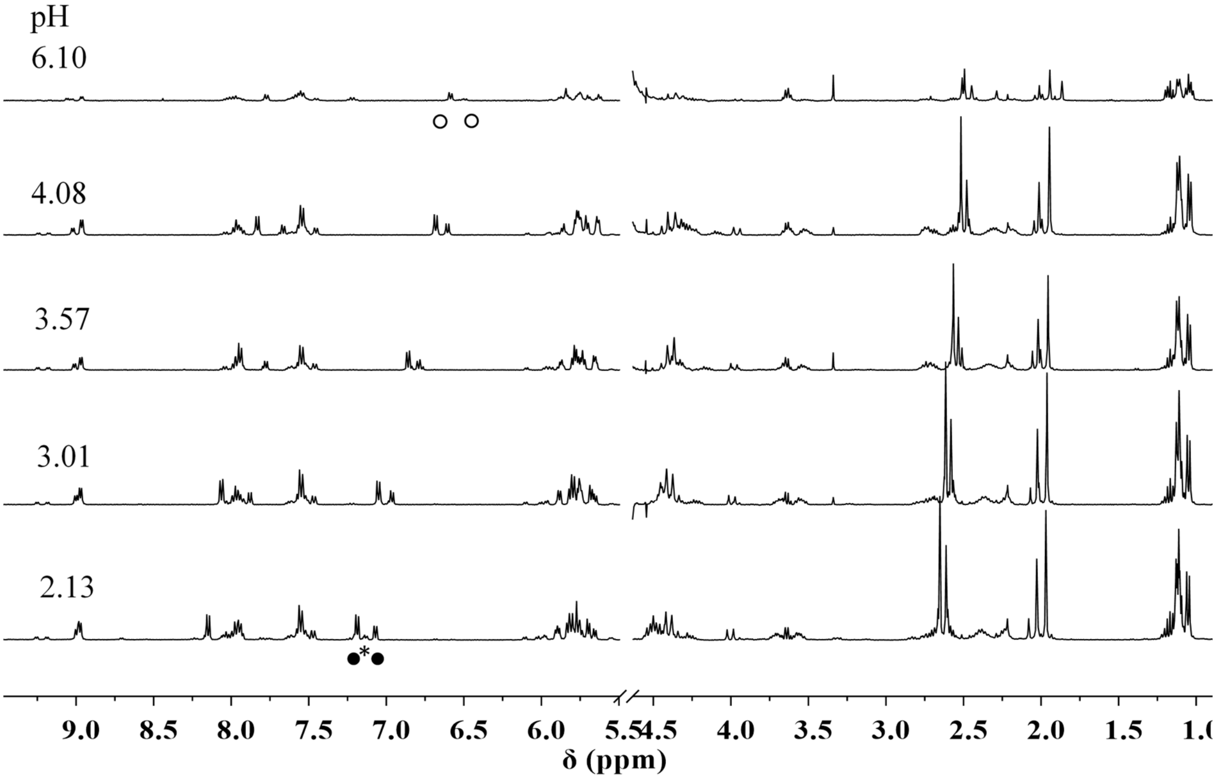

Figure S18. pH-dependence of the signals belonging to the protons in the[(η^6-*p*-cym)Ru]$^{2+}$–L5 system at 1:1 ratio, c_{L5} = 5.0 mM, t = 48 h. Symbols represent the signals of the following species: H_x(L5) (*), [M(H_2O)$_3$]$^{2+}$ and [M(Cl)$_3$]$^-$ (#), [MH_2L]$^{3+}$ (•), [MHL]$^{2+}$ (○).

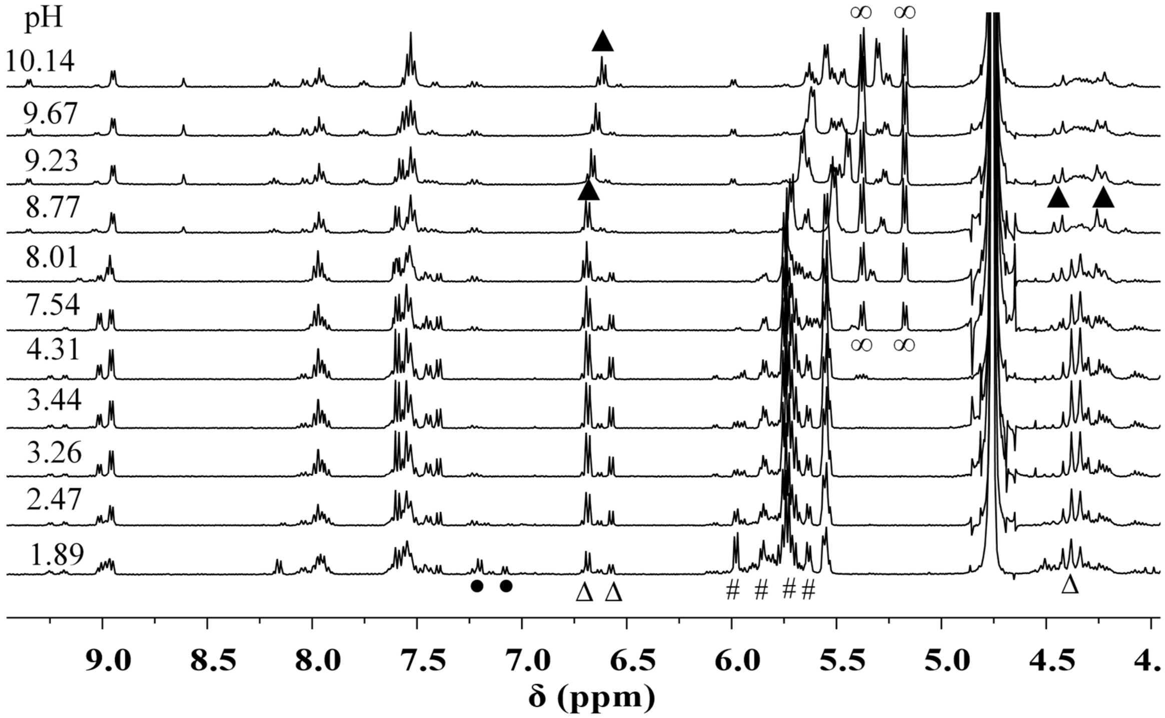

Figure S19. pH-dependence of the signals belonging to the protons in the [(η^6-*p*-cym)Ru]$^{2+}$–L5 system at 2.2:1 ratio, c_{L5} = 5.0 mM. Symbols represent the signals of the following species: $H_x(L5)$ (*), $[M(H_2O)_3]^{2+}$ and $[M(Cl)_3]^-$ (#), $[MH_2L]^{3+}$ (•), $[M_2L]^{3+}$ (Δ), $[M_2H_{-1}L]^{2+}$ (▲), $[M_2(OH)_3]^+$ (∞).

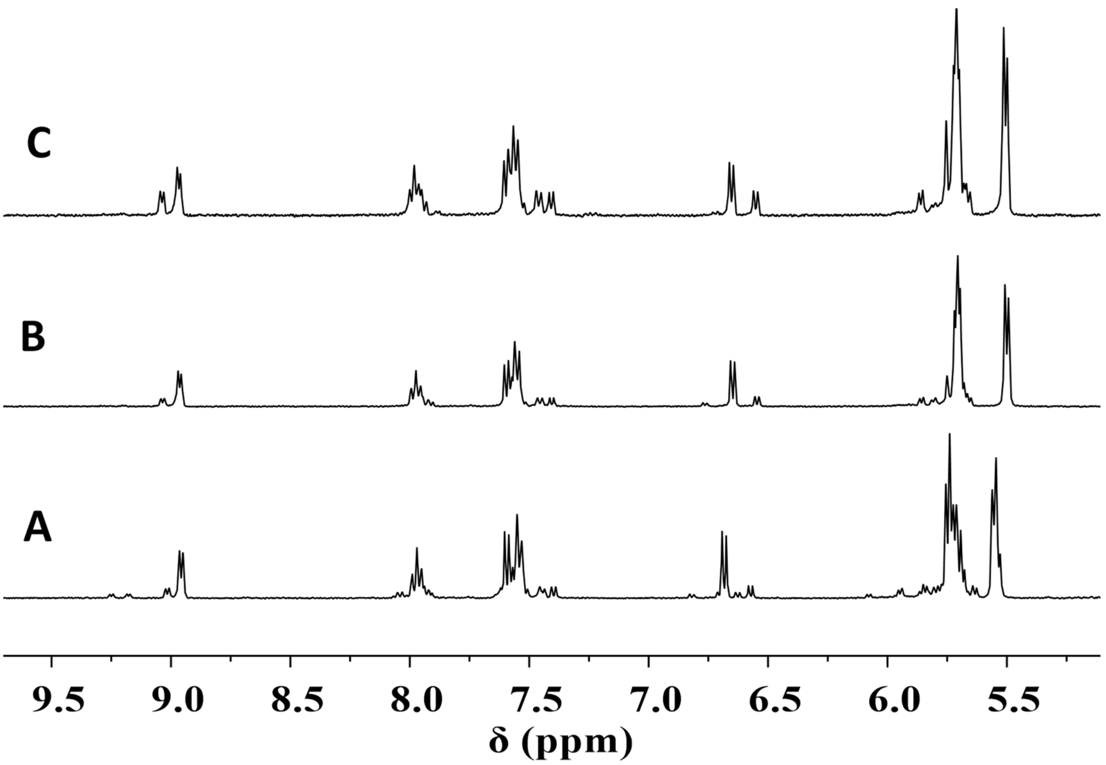

Figure S20. Dependence on time and chloride ion concentration of the low field NMR spectra registered in the [(η[6]-*p*-cym)Ru]$^{2+}$–L5 system at 2:1 metal ion to ligand ratio (c_{L5} = 5 mM). A: I = 0.20 M (KNO_3); pH = 3.74; t = 0 h. B: I = 0.20 M (KNO_3) + 0.20 M (KCl); pH = 3.74; t = 0 h. C: I = 0.20 M (KNO_3) + 0.20 M (KCl); pH = 3.74; t = 24 h.
